# Predicting future biomass yield in *Miscanthus* using the carbohydrate metabolic profile as a biomarker

**DOI:** 10.1111/gcbb.12418

**Published:** 2017-01-21

**Authors:** Anne L. Maddison, Anyela Camargo‐Rodriguez, Ian M. Scott, Charlotte M. Jones, Dafydd M. O. Elias, Sarah Hawkins, Alice Massey, John Clifton‐Brown, Niall P. McNamara, Iain S. Donnison, Sarah J. Purdy

**Affiliations:** ^1^ Institute of Biological, Environmental and Rural SciencesAberystwyth UniversityPlas GogerddanSY23 3EBUK; ^2^ Centre for Ecology and HydrologyLancaster Environment CentreLibrary AvenueBailriggLancasterLA1 4APUK

**Keywords:** ^13^C, bioenergy, biomarkers, carbohydrates, cell wall, *Miscanthus*, soluble sugars, starch

## Abstract

In perennial energy crop breeding programmes, it can take several years before a mature yield is reached when potential new varieties can be scored. Modern plant breeding technologies have focussed on molecular markers, but for many crop species, this technology is unavailable. Therefore, prematurity predictors of harvestable yield would accelerate the release of new varieties. Metabolic biomarkers are routinely used in medicine, but they have been largely overlooked as predictive tools in plant science. We aimed to identify biomarkers of productivity in the bioenergy crop, *Miscanthus,* that could be used prognostically to predict future yields. This study identified a metabolic profile reflecting productivity in *Miscanthus* by correlating the summer carbohydrate composition of multiple genotypes with final yield 6 months later. Consistent and strong, significant correlations were observed between carbohydrate metrics and biomass traits at two separate field sites over 2 years. Machine‐learning feature selection was used to optimize carbohydrate metrics for support vector regression models, which were able to predict interyear biomass traits with a correlation (*R*) of >0.67 between predicted and actual values. To identify a causal basis for the relationships between the glycome profile and biomass, a ^13^C‐labelling experiment compared carbohydrate partitioning between high‐ and low‐yielding genotypes. A lower yielding and slower growing genotype partitioned a greater percentage of the ^13^C pulse into starch compared to a faster growing genotype where a greater percentage was located in the structural biomass. These results supported a link between plant performance and carbon flow through two rival pathways (starch vs. sucrose), with higher yielding plants exhibiting greater partitioning into structural biomass, via sucrose metabolism, rather than starch. Our results demonstrate that the plant metabolome can be used prognostically to anticipate future yields and this is a method that could be used to accelerate selection in perennial energy crop breeding programmes.

## Introduction

*Miscanthus* is a candidate lignocellulosic biofuel crop owing to its high productivity and low chemical input requirements (Visser & Pignatelli, 2001; Somerville *et al*., 2010). As a C4 grass, it is a close genetic relative of two major biofuel crops, *Zea mays* (maize) and *Saccharum* Sp. (sugarcane; Hodkinson *et al*., 2002). However, currently, the only commercially grown genotype of *Miscanthus* is a wild accession and not a breeder's line. Therefore, several breeding programmes are now targeting *Miscanthus* for yield and quality improvement. A major hindrance to the improvement of perennial energy crops through breeding is the long duration for new crosses to reach maturity when they can be assessed for superiority (Purdy *et al*., 2015). In *Miscanthus*, this is typically in the region of 4 years from when a seed is planted. There is a pressing need to identify new methods to accelerate the selection of elite crosses in *Miscanthus* and other perennial species.

In plant science, numerous studies have demonstrated associations between metabolites and various stress conditions such as increases in proline during chilling (Wanner & Junttila, 1999) or increases in jasmonic acid in response to herbivory (Wang & Wu, 2013). In medicine, metabolic biomarkers are used prognostically, that is to anticipate a future outcome in an asymptomatic individual, an example being the measure of blood cholesterol as a predictor of future heart attack risk. However, in plant science, the metabolome has rarely been used to predict future outcomes in crop species (Steinfath *et al*., 2010). In *Arabidopsis thaliana,* several studies have successfully correlated biomass with particular metabolites, groupings of metabolites and enzyme activities (when expressed against total protein content; Meyer *et al*., 2007; Sulpice *et al*., 2009, 2010, 2013; Scott *et al*., 2014). By combining a negative correlation with starch and a positive correlation with enzyme activities, approximately a third of the variation in biomass of an *Arabidopsis* inbred family could be accounted for (Sulpice *et al*., 2010). A notable example of biomarker identification in a crop species is in potato, where the abundance of glucose and fructose was found to positively correlate with discoloration during frying (low chip quality). When either of these hexoses was used as markers to predict chip quality in new crosses, the correlation (*R*_S_) between predicted and measured quality was 0.67 (Steinfath *et al*., 2010). In a recent study into drought tolerance in rainforest trees, the abundance of nonstructural carbohydrates (NSC) was found to positively correlate with drought tolerance in trees showing natural variation and in those that had been manipulated (O'Brien *et al*., 2014). These studies show that metabolites can be used as biomarkers to predict biomass, quality traits and stress responses in species as diverse as *Arabidopsis*, potato and rainforest trees. In all these studies, it was carbohydrates that were successfully used as markers.

We recently showed that two fast‐growing and high‐yielding genotypes of the perennial bioenergy grass, *Miscanthus*, displayed a distinctive NSC profile compared to two slower growing genotypes and that this phenotype was consistent across 2 years and different environments (Purdy *et al*., 2015). However, the limited number of genotypes and hybrids used in this study were insufficient to unequivocally determine whether the carbohydrate metabolic profile (‘glycome’) could be used as a biomarker of productivity. The phenotypic attribute so far shown to most strongly correlate with final yield is (log‐transformed) maximum canopy height (*R* ^2^ = 0.55; Robson *et al*., 2013). Therefore, our primary aim with this study was to identify single or multiple metabolic biomarkers that could predict yield in *Miscanthus* and to determine how the strength of the correlations compared with height as a predictor. *Miscanthus* is usually harvested at the end of winter when senescence is complete, but we sampled carbohydrates in stems in the middle of UK summer when growth was most rapid. The summer carbohydrate metabolic profile was then used to predict winter yields harvested the following year.

The choice of nonstructural carbohydrates to profile was based on previously observed genotypic differences in abundance and partitioning in four genotypes (Purdy *et al*., 2014, 2015). Sucrose is the most abundant soluble sugar in *Miscanthus,* and owing to the close phylogenetic relationship between *Miscanthus* and sugarcane (Hodkinson *et al*., 2002), it was an obvious candidate for study in diverse genotypes. Sucrose is formed of a molecule each of glucose and fructose, and relationships between the hexoses and biomass traits had previously been observed (Purdy *et al*., 2015). Unlike many C3 temperate grasses, C4 species such as *Miscanthus* do not accumulate fructans (Muguerza *et al*., 2013) but instead accumulate starch as a transient form of storage carbohydrate (de Souza *et al*., 2013; Purdy *et al*., 2014).

To grow, plants must accumulate structural mass, predominantly cellulose and the cell wall hemicellulose polysaccharides. Both starch and cellulose are polymers of glucose, and we hypothesized that rapidly growing genotypes of *Miscanthus* may be accumulating cellulose more rapidly at the expense of starch biosynthesis, thus explaining the negative relationship between starch and growth observed in our previous study and that of others (Rocher, 1988; Sulpice *et al*., 2009; Purdy *et al*., 2014, 2015). Therefore, starch, cellulose and the hemicelluloses were also assayed to assess a potential role as yield biomarkers.

## Materials and methods

### Mixed population

A total of 244 *Miscanthus* genotypes were collected and planted as described previously (Allison *et al*., 2011; Jensen *et al*., 2011; Robson *et al*., 2012). From this population, a selection of seven short and 11 tall plants were used in the experiment. A description of the different species is provided in Table 1. Three biological replicates per genotype were harvested from blocks 1, 2 and 3 of the trial.

**Table 1:** The species and experimental structure of the mixed population and the mapping family

Species	*n* Total	*n* Tall	*n* Short
*Miscanthus* mixed population			
*M. sinensis*	10	5	5
Hybrid	4	4	0
*M. sacchariflorus*	4	2	2
*Miscanthus* mapping family			
*M. sinensis*	1	1	0
Hybrid	19	9	10

### Mapping family

A total of 102 genotypes from a paired cross between a diploid *M. sinensis* and a diploid *M. sacchariflorus* were sown from seed in trays in a glasshouse in 2009. In 2010, individual plants were split to form three replicates of each genotype and then planted out into the field in a spaced‐plant randomized block design comprising three replicate blocks. The field site is located 300 m to the south from the mixed population (described above), and therefore, stone content and soil types are as described previously (Allison *et al*., 2011); however, the field containing the mapping family is on a gentler slope than the mixed population.

### Biomass trait measurements

Growth rate: Canopy heights of the selected plants were measured weekly. The values presented are for the 2‐week period surrounding the harvests to give a value of growth rate cm day^−1^.

Stem height: A single stem that was representative of canopy height was selected for destructive harvest and its height (cm) measured on the day of harvest.

### Destructive harvests

A single stem that was representative of canopy height was selected from each plant, cut at a height of 10 cm from the base, measured then flash‐frozen before freeze‐drying. As NSC show diurnal fluctuations in *Miscanthus* (Purdy *et al*., 2013), the two sets of plants were harvested on different days so that each harvest could be completed within a 2‐h window at the same time of day (Zt 8–10 of a 16‐h photoperiod). The mixed population was harvested on 04 July 2013, and the mapping family was harvested on 19 July 2013. For the harvesting of the entire mapping family in 2014, each of the three blocks were harvested on consecutive days in July to stay within the 2‐h time window specified above.

Annual yield harvest: The mixed population and mapping family were destructively harvested for yield in March 2014 (following the 2013 growing season), and the mapping family was harvested in Feb 2015 following the 2014 growing season. Biomass was dried to a constant weight, and then, the average DW weight per plant (kg) was calculated.

### Nonstructural carbohydrate (NSC) compositional analyses

Soluble sugars and starch were analysed as previously described (Purdy *et al*., 2014, 2015). Soluble sugar extraction: approximately 20 mg (actual weight recorded) of each cryomilled (6870 Freezer Mill, Spex, Sampleprep, Stanmore, UK) plant tissue sample was weighed into 2‐mL screwcap microcentrifuge tubes. Sugars were extracted four times with 1 mL of 80% (v/v) ethanol and the resulting supernatants pooled; two extractions were at 80 °C for 20 min and 10 min, respectively, and the remaining two at room temperature. A 0.5 mL aliquot of soluble sugar extract and the remaining pellet containing the insoluble fraction (including starch) were dried down in a centrifugal evaporator (Jouan RC 1022, Saint‐Nazaire, France) until all the solvent had evaporated. The dried down residue from the soluble fraction was then resuspended in 0.5 mL of distilled water. Samples were stored at −20 °C for analysis.

Soluble sugar analysis: Soluble sugars of samples extracted in the previous step were quantified enzymatically by the stepwise addition of hexokinase, phosphoglucose isomerase and invertase (Jones *et al*., 1977). Samples were quantified photometrically (Ultraspec 4000; Pharmacia Biotech, Uppsala, Sweden) by measuring the change in wavelength at 340 nm for 20 min after the addition of each enzyme. Sucrose, glucose and fructose were then quantified from standard curves included on each 96‐well plate.

Starch quantification: Starch was quantified using a modified Megazyme protocol (Megazyme Total Starch Assay Procedure, AOAC method 996.11; Megazyme International, Wicklow, Ireland). Briefly, the dried pellet was resuspended in 0.4 mL of 0.2 m KOH, vortexed vigorously and heated to 90 °C in a water bath for 15 min to facilitate gelatinization of the starch. A total of 1.28 mL of 0.15 m NaOAc (pH 3.8) was added to each tube (to neutralize the sample) before the addition of 20 μL *α*‐amylase and 20 μL amyloglucosidase (Megazyme International). After incubation at 50 °C for 30 min and centrifugation for 5 min, a 0.02 mL aliquot was combined with 0.6 mL of GOPOD reagent (Megazyme). A total of 0.2 mL of this reaction was assayed photometrically (Ultraspec 4000; Pharmacia Biotech) on a 96‐well microplate at 510 nm against a water‐only blank. Starch was quantified from known standard curves on the same plate. Each sample and standard was tested in duplicate. Each plate contained a *Miscanthus* control sample of known concentration for both soluble sugars and starch analysis.

### Cell wall carbohydrates and lignin

Lignin and matrix polysaccharides were analysed as described by Foster *et al*. (2010a,b). To quantify matrix polysaccharides, a Dionex ICS‐5000DC (Thermo Scientific, Loughborough, UK) was used. Each chromatographic run contained sets of standards and a dilution series. Lignin quantification followed the method described by Foster *et al*. (2010a). Crystalline cellulose was analysed by Seaman hydrolysis and subsequent quantification of glucose (Purdy *et al*., 2014).

### Crystalline cellulose

Approximately 60 mg (actual weight recorded) of purified cell wall was hydrolysed with 0.6 mL of 72% H_2_SO_4_, vortexed and left to incubate whilst shaking at 200 rpm for 1 h at 30 °C. After incubation, samples were diluted with 16.8 mL of deionized H_2_O. Tubes were then capped and autoclaved at 121 °C for 1 h. Once cooled, an aliquot of 0.65 mL was neutralized with 30 mg CaCO_3_ and centrifuged to pellet the CaCO_3_ and the supernatant was removed to a fresh tube. Glucose was quantified enzymatically as previously described (Purdy *et al*., 2014). Standards of glucose were treated alongside experimental samples and included on each plate with a duplicated check sample.

The amount of glucose that was derived from hemicellulose (as described above) was subtracted from the total to give a value derived just from the Seaman hydrolysis of crystalline cellulose.

### Data modelling

Principal component analysis (PCA) was performed in simca‐p v.11 (Umetrics AB, Malmo, Sweden) on values averaged across all biological replicates (usually three) of each genotype in each sampled population. Data were mean‐centred and scaled to unit variance, and the reported PC was ‘significant’ in the default simca‐p cross‐validation procedure. Machine learning employed the *AttributeSelectedClassifier* in the Explorer interface of weka v.3.6 (Frank *et al*., 2004). This weka ‘metaclassifier’ firstly sought an optimal subset of biomass predictors from the individual carbohydrate levels and their derivative metrics (i.e. sums and ratios). This involved testing all potential predictor combinations by the *ExhaustiveSearch* algorithm with evaluation by *CfsSubsetEval*. The metaclassifier then trained a support vector regression algorithm, *SMOreg*, to relate the selected carbohydrate predictors to their associated biomass trait data. The resultant model was evaluated for its accuracy in predicting the relevant biomass values from the carbohydrate metrics of a ‘test’ set of plants. Test data were always completely excluded from model training. Default parameters were used for each algorithm.

### ^13^CO_2_ pulse labelling

Our chamber design and ^13^C pulse‐labelling approach were similar to previous methods (Hogberg *et al*., 2008; Subke *et al*., 2009; Biasi *et al*., 2012) and applied to a field trial planted in 2010 with triplicate plots of a *M. sinensis*, a *M. sinensis* × *M. sacchariflorus* hybrid (*M. x giganteus*) and a *M. sacchariflorus* in a randomized block design. In each replicate plot, square ^13^C pulse chambers were erected (2 m *l*, 2 m *w*, 3 m *h*) above the crop, resulting in a total tent volume of 12 m^3^. Aluminium scaffold was used to support plastic polythene film, which allowed 90% of photosynthetically active radiation to enter the chamber. During the ^13^C pulse, the chamber was sealed at the base. To counter ambient air temperature increases within the chambers, each was cooled using a water cooled, split air conditioner (Andrew Sykes, Wolverhampton, UK).

The ^13^C pulse labelling was carried out on 23 July 2013 at ca. 08:20 by introducing ca. 6 L of 99% ^13^C‐atom enriched pure CO_2_ (CK Gases, Ibstock, UK) in sequential batches after sealing the tent.

### ^13^C Harvesting and sample preparation

Pulsed samples were harvested 30 h after labelling. A single marked stem was harvested as previously described. The level of ^13^C enrichment above natural background ^13^C levels was determined in the soluble, starch and structural mass, which was extracted as previously described.

Solid sample analysis was performed on a Costech EC S4010 Elemental Analyser (Costech Analytical Technologies Inc, Valencia, CA, USA) coupled to a Picarro G‐2131i Series CRDS analyzer (Picarro Inc, Santa Clara, CA, USA) via a split‐flow interface using a method similar to (2013). Cryomilled samples of ~2 mg were weighed into ultraclean, 6 × 4 mm pressed tin cups (Elemental MicroAnalysis Ltd, Okehampton, UK), crimped and loaded into a Zero N‐Blank, 50 position carousel, autosampler. From the autosampler, samples were dropped at a throughput of 1 every 15 min into a combustion reactor, maintained at a constant 980 °C. Samples undergo flash combustion and thermally decompose. Evolved CO_2_ was passed through a thermal conductivity detector (TCD) for C detection and then vented through a split‐flow interface to the Picarro CRDS analyzer for ^13^C analysis. Standard materials covering a representative range of C and *δ* ^13^C values were run during each analysis batch, and results were calibrated against these.

### ^13^C Calculations

#### Stable isotope notation

Studies of this kind have generally either expressed ^13^C enrichment values in *δ* ^13^C, a measure of the ratio of ^13^C and ^12^C, reported in parts per thousand (‰) relative to a standard value (Pee Dee Belemnite – PDB) or atom %. Outputs from the Picarro ^13^CO_2_ analyzer were in standard delta (*δ*) value notation (*δ* ^13^C). *δ* ^13^C values are calculated using the following equation:

(1) $$\delta^{13}C\;\text{sample}=\frac{{}^{13}C{/}^{12}C\;\text{sample}}{{}^{13}C{/}^{12}C\;\text{PDB}}-1\times 1000,$$

where ^13^C/^12^CPDB is the isotopic ratio of the standard material PDB given as 0.0112372. Results were converted to atom % and then mg g^−1^ using the following equations:

(2) $$\text{Atom}\;\%=\frac{100\times \text{AR}\times (\delta^{13}C/1000+1)}{1+\text{AR}\times (\delta^{13}C/1000+1)},$$

where AR = 0.011237. The absolute ratio of standard material (PDB) and *δ* ^13^C = standard delta value of sample.

(3) $$\text{mg}\;g^{-1}=\text{Atom}\;\%\times 10.$$

### Statistical analyses

Differences between genotypes for biomass traits, NSC, structural carbohydrates and block effects were determined from one‐way anova using genotype or block as the treatment factor (*P* =≤ 0.05). Genotypic differences in the deposition of ^13^C were determined by one‐way anova using genotype as the treatment factor and an associated Tukey's HSD test. anova, Tukey and Wilcoxon tests were performed using genstat (13th Edition). Differences between genotypes grouped as ‘tall’ or ‘short’ were determined from Student's two‐tailed *t*‐tests (assuming unequal variance; *P* =≤ 0.05) using Microsoft Excel. To compare biomass measures and NSC between across years (2013 and 2014), a Pearson correlation was performed to determine similarities in absolute values and a Spearman rank correlation analysis to compare the ordering of genotypes. Both analyses were carried out in sigmaplot 12 (Systat Software, Inc, San Jose, CA, USA).

## Results

Two sets of field‐grown plants were studied (Table 1). The ‘mixed population’, from which 18 plants were selected for study in 2013 (their eighth growing season), was comprised of *M. sinensis*,* M. sinensis* × *M. sacchariflorus* hybrids and *M. sacchariflorus* genotypes. The ‘mapping family’, from which 20 plants were selected for study in 2013 (their fourth growing season), was comprised of *M. sinensis* × *M. sacchariflorus* hybrids, plus a single, tall, *M. sinensis* genotype. Nonstructural and structural carbohydrates and lignin were sampled in July, during the summer growing season. Measures of biomass traits obtained during the summer sampling were stem height, and growth rate over the surrounding two‐week period, whereas annual yield was obtained at harvest after the following winter. Carbohydrate and biomass data for all genotypes are in Tables S1–S3.

Height has been shown to be the trait that best correlates with final yield in *Miscanthus* (Robson *et al*., 2013). Therefore, each set of plants was divided into ‘tall’ and ‘short’ classes for comparison of carbohydrate contents. The average heights of plants grouped as short or tall from the mixed population were 79 cm and 151 cm, respectively, and in the mapping family, the average heights of the short and tall classes were 56 cm and 120 cm, respectively (Table S1).

**Figure 1:**
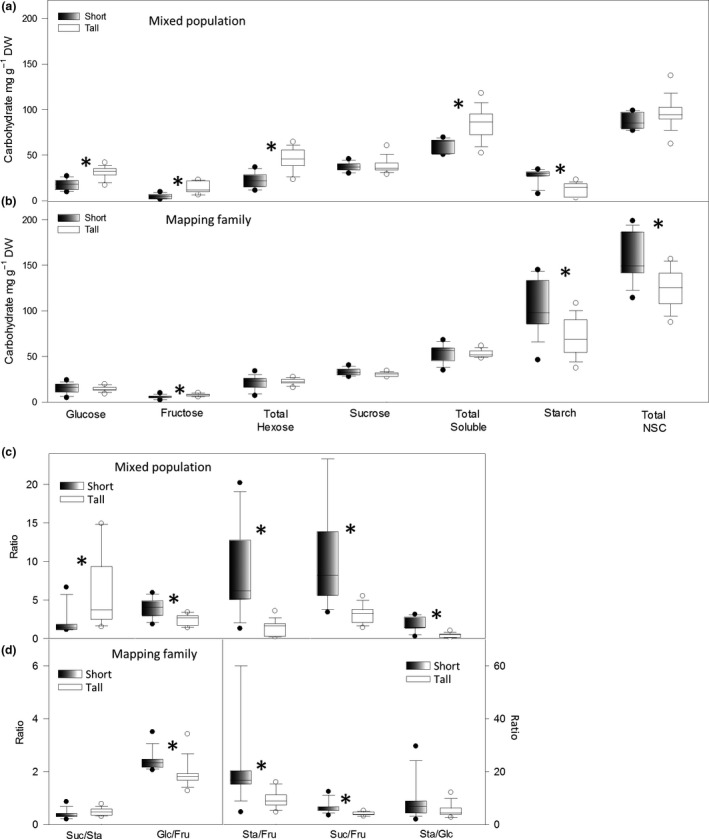
Quantification of NSC in short and tall genotypes of the mixed population (a) and mapping family (b), and ratios between carbohydrate levels in short and tall genotypes of the mixed population (c) and mapping family (d). Circles show outliers. Significant differences between tall and short plants are shown by an asterisk (Student's *t*‐test assuming unequal variances, *P* =≤ 0.05). Key: NSC = total nonstructural carbohydrate, Suc/Sta = sucrose‐to‐starch ratio, Glc/Fru = glucose‐to‐fructose ratio, Sta/Fru = starch‐to‐fructose ratio, Suc/Fru = sucrose‐to‐fructose ratio, Sta/Glc = starch‐to‐glucose ratio.

In both sets of plants, the abundance of fructose was significantly greater in the tall plants compared to the short plants, whereas the opposite was true for starch, which was significantly more abundant in the short plants (Fig. 1a and b; Table S2). Glucose, total hexose and total soluble carbohydrates were significantly different between tall and short plants only in the mixed population.

The ratios between different NSC were examined in the short and tall groups, to further investigate the contrasting relationships of fructose and starch with plant height. The greatest difference between tall and short plants was the starch/fructose ratio, which was >fourfold greater in the short plants of the mixed population and >twofold greater in the short plants of the mapping family (Fig. 1c and d). The glucose/fructose and sucrose/fructose ratios were also significantly negatively associated with height in both populations. Differences in the starch/glucose and sucrose/starch ratios of tall and short plants were significant only in the mixed population (Fig. 1c and d).

**Figure 2:**
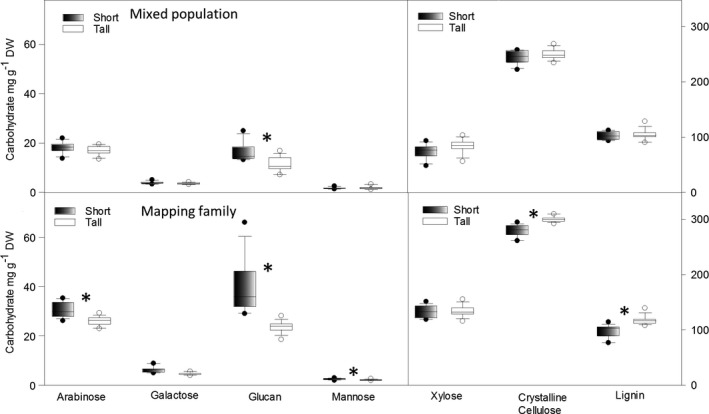
Quantification of structural carbohydrates and lignin in short and tall genotypes of the mixed population (a) and mapping family (b). Circles show outliers. Significant differences between tall and short plants are shown by an asterisk (Student's *t*‐test assuming unequal variances, *P* =≤ 0.05).

Significant differences in hemicellulosic glucan were observed between the short and tall plants in both populations, with short plants exhibiting higher glucan levels (Fig. 2a and b). Other significant differences, seen only in the mapping family, were arabinose, galactose and mannose, which were more abundant in short plants, whereas crystalline cellulose and lignin were more abundant in tall plants (Fig. 2b; Table S3b). In the mixed population, these last cell wall components did not show significant height‐associated differences (Fig. 2a; Table S3a), possibly due to a significant effect of the replicate field blocks at the relevant trial site (Table S4). These replicate blocks were arranged parallel to the slope of the hill on which the mixed population was grown (see *Materials and Methods*). A block effect was previously reported in this population at the end of the growing season and attributed to differences in wind exposure and water dynamics (Allison *et al*., 2011). No significant block effects were detected for any of the NSC from either set of plants, or in the cell wall composition of the mapping family (Table S4).

**Figure 3:**
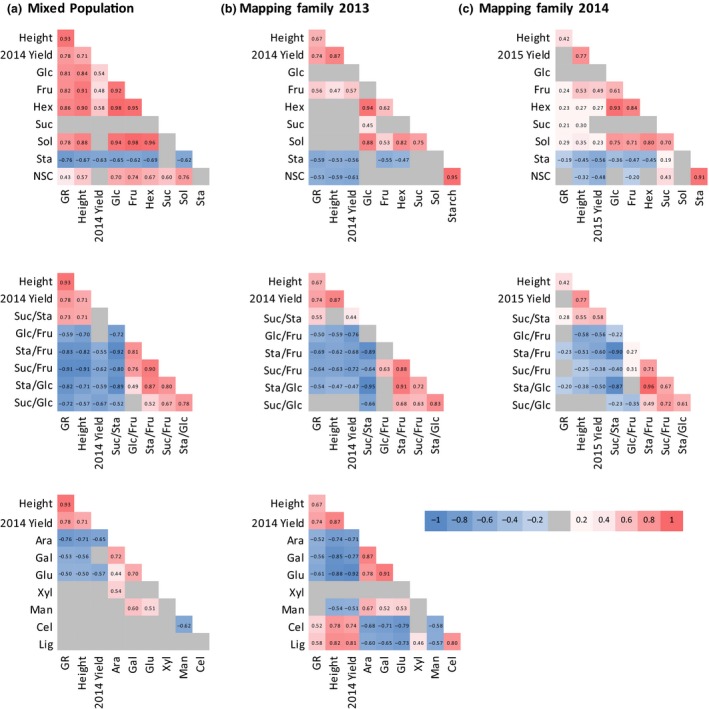
Spearman's rank correlations between biomass parameters, NSC levels (top) and ratios (middle), and cell wall components (bottom) in the Miscanthus mixed population in 2013 (a) and the mapping family in 2013 (b) and 2014 (c). Significant positive correlations are coloured red, and significant negative correlations are coloured blue (*P* =≤ 0.05). Nonsignificant correlations are coloured grey. Key: Glc = glucose, Fru = fructose, Hex = total hexose, NSC = total nonstructural carbohydrate, Suc = sucrose, Sta = starch, Glc/Fru = glucose‐to‐fructose ratio, Suc/Glc = sucrose‐to‐glucose ratio, Suc/Fru = sucrose‐to‐fructose ratio, Suc/Sta = sucrose‐to‐starch ratio, Sta/Glc = starch‐to‐glucose ratio, Ara = arabinose, Cel = cellulose, Glu = glucan, Gal = galactose, Lig = lignin.

Having found differences in carbohydrates of height classes of plants selected in 2013, we examined the potential for using carbohydrates to model biomass traits (summer height, summer growth rate and annual yield). To provide more data for this purpose, a much larger selection (102 genotypes) was made in 2014 from the mapping family and similarly analysed for NSC and biomass traits. As an exploratory stage for modelling, we first sought to correlate carbohydrate contents with biomass traits in each set of plants, using Spearman's rank coefficients (*R*_S_), which are robust to potential nonlinearity. Of the NSC in the mixed population, both glucose and fructose produced positive correlations with biomass traits, with the strongest correlation being between fructose and stem height (0.91; Fig. 3a). In the mapping family, biomass traits were significantly positively correlated with fructose (correlation coefficients of ~0.5), though not with glucose (Fig. 3b and c). No strong correlation between sucrose and biomass traits was detected in either set (Fig. 3a–c). In contrast, starch negatively correlated with all biomass traits (and with fructose) in both sets of plants (Table S3a–c), the strongest relationship being with growth rate in the mixed population (−0.76). The relationships observed in the mapping family in 2013 and in the extended number of genotypes in 2014 were largely consistent, both showing strong positive correlations of fructose with stem height and yield (~0.5) and strong negative relationships between starch and yield (Fig. 3b and c). Correlations with growth rate, however, were considerably lower in 2014 than 2013 (Fig. 3b and c).

Ratios between NSC produced stronger correlations with biomass traits than did the individual components (Fig. 3a–c, middle row). In both the mixed population and mapping family, significant negative correlations between biomass traits and the starch/fructose and sucrose/fructose ratios were observed. Conversely, positive correlations between the sucrose/starch ratio and biomass traits were observed in both sets of plants (Fig. 3a–c).

In both sets of plants, the matrix polysaccharide components arabinose, galactose and glucan were negatively correlated with biomass traits (Fig. 3a and b, bottom row). The strongest negative correlation in the mixed population was between arabinose and growth rate (−0.76), whilst in the mapping family, matrix glucan correlated negatively with yield (−0.92). In the mapping family, crystalline cellulose and lignin showed positive correlations with biomass traits of 0.5–0.8, whereas these relationships were not observed in the mixed population (Fig. 3a and b).

**Figure 4:**
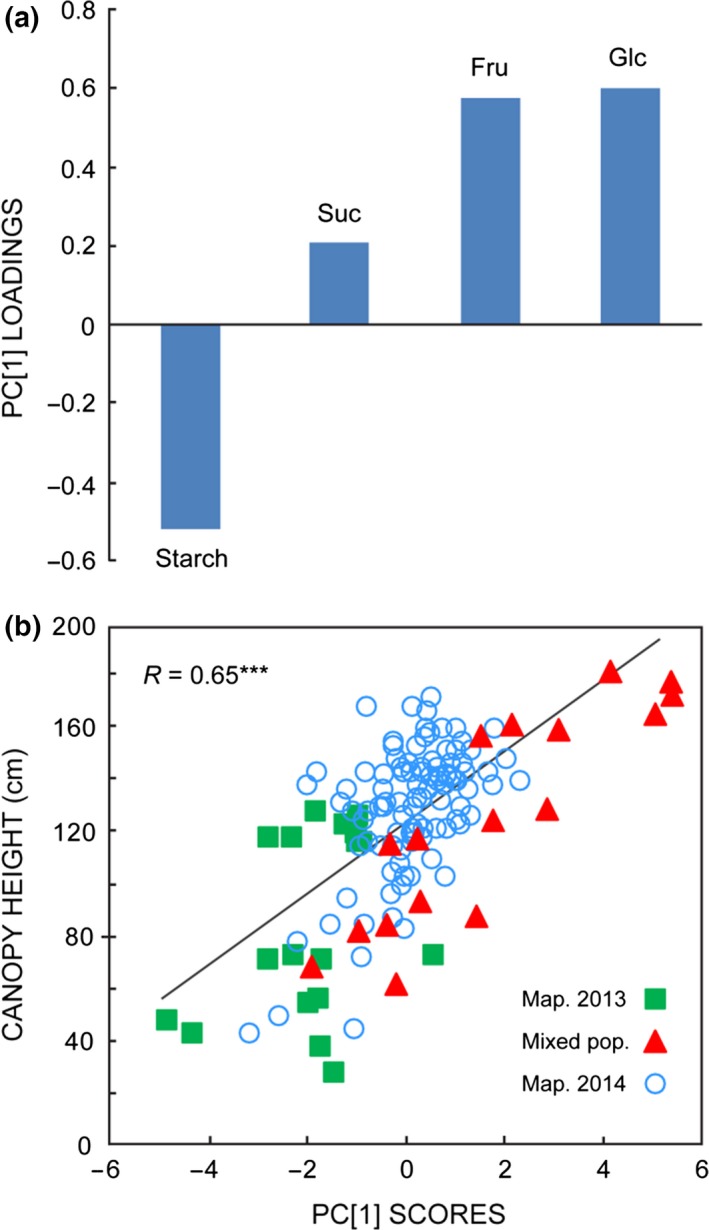
Correlation of multivariate carbohydrate phenotypes and biomass across all experimental populations. Horizontal axis shows scores on PC[1], the major component (58% of variance) from PCA of NSC levels (Glc, Fru, Suc, starch) of each genotype in the mixed population (triangles), the mapping population selected in 2013 (squares) and the extended mapping family analysed in 2014 (open circles). Vertical axis shows mean canopy height of each sampled genotype. *R* indicates Pearson correlation (***, *P* < 0.001) between heights and PC[1] scores.

As similar correlations manifested in the various genotypes, in two different field trial sites and two different years (Fig. 3), it was pertinent to ask whether a ‘carbohydrate phenotype’ related to biomass traits was sufficiently robust to be identifiable across all sampled populations. When the NSC levels (glucose, fructose, sucrose, starch) measured in all populations were subjected to PCA in a unified data set, the first component, PC[1], accounted for 58.3% of overall variance (Fig. 4). The loadings for each NSC on PC[1] identified opposite variations in starch on the one hand, and the hexoses fructose and glucose on the other (Fig. 4a). Thus, PC[1] could be regarded as a composite index of NSC status, such that negative scores on PC[1] were indicative of high starch and low hexoses, and the converse for positive PC[1] scores. Moreover, PC[1] scores of the sampled genotypes showed significant correlations to biomass traits. As seen in Fig. 4b, genotypes with negative PC[1] scores (high starch, low hexoses) tended to be characterized by shorter stature than genotypes with positive scores (low starch, high hexoses). Pearson correlations with PC[1] scores were highly significant (*P* < 10^−12^) for height (*R*, 0.65), yield (*R*, 0.56) and growth rate (*R*, 0.62).

Considering the carbohydrate/biomass correlations in different *Miscanthus* populations (Fig. 3), and evidence for a biomass‐correlated multivariate ‘carbohydrate phenotype’ across populations (Fig. 4), we sought to test the predictive power of the glycome for biomass traits by multivariate modelling. A machine‐learning approach was chosen to address the complexity of the potentially relevant carbohydrate and genetic data, in the context of the small number and variability of biological replicates typical of a screening trial in the field.

As evident from Fig. 3, a number of potential carbohydrate metrics, including sums and ratios of individual NSC, would be available for inclusion in such a model. However, use of too many of these metrics would be redundant. We therefore used machine learning to identify parsimonious subsets of effective predictors from the following list of 20: glucose, fructose, total hexose, sucrose, total soluble carbohydrates, starch, total NSC, arabinose, galactose, glucan, xylose, mannose, crystalline cellulose, lignin and the ratios sucrose/starch, glucose/fructose, starch/fructose, sucrose/fructose, starch/glucose and sucrose/glucose. For the full list of 20 carbohydrate predictors, 1 048 576 possible combinations needed evaluation. We also fitted models using only the NSC predictors (8192 combinations), and excluding starch, only soluble sugar predictors (256 combinations). Evaluations were performed using a ‘correlation‐based feature selection’ algorithm, *CfsSubsetEval*, which prefers sets of predictors that have low correlation amongst themselves, but each has high predictive worth (Wang *et al*., 2005).

A support vector regression algorithm, *SMOreg*, was ‘trained’ to fit regression models of a given biomass trait using the NSC predictors selected in the above process. Support vector machines were originally developed for classification and may be conceived as mapping to higher dimensional space of a ‘hyperplane’ between two data classes, the ‘support vectors’ being the least‐separated members of the opposite classes. To perform regression, the task is converted to classification by duplicating each *y*‐axis value by addition and subtraction of a new parameter *ε*. The hyperplane between the plus‐*ε* and minus‐*ε* ‘classes’ is equivalent to a regression function (Li *et al*., 2009). Regression modelling was investigated for each *Miscanthus* population by cross‐validation, in which model performance was averaged over nine successive random partitions of the data into training and validation subsets. For each partition, both predictor selection and *SMOreg* modelling used the training data, and models were tested on the held‐out validation data (Table 2).

**Table 2:** Machine‐learning models of biomass traits using carbohydrate data

A. Canopy height		Information included in models		
		Genotype, carbohydrates	Carbohydrates only	
		All genotype replicates	All genotype replicates	Averaged by genotype
Plants	Carbohydrate fractions	*R* values		
Mixed population	All	0.92***	0.44***	0.76***
	Nonstructural	0.81***	0.70***	0.81***
	Soluble	0.84***	0.52***	0.70**
Mapping family 2013	All carbohydrates	0.94***	0.72***	0.77***
	Nonstructural	0.92***	0.63***	0.68***
	Soluble	0.93***	0.68***	0.70***
Mapping family 2014	Nonstructural	0.88***	0.61***	0.76***
	Soluble	0.86***	0.44***	0.66***
Carbohydrate fractions	Constituents common to predictors of all models^a^			
All	**Glucan**;** Fructose** (as Fru, Glc/Fru, Hex or Suc/Fru); **Glucose** (as Glc/Fru, Hex or Sta/Glc); **Starch** (as Sta, Sta/Glc or Suc/Sta)			
Nonstructural	**Fructose** (as Fru, Glc/Fru, Hex, NSC or Suc/Fru); **Glucose** (as Glc/Fru, Hex, NSC, Sta/Glc or Suc/Glc); **Starch** (as NSC, Sta, Sta/Fru, Sta/Glc or Suc/Sta)			
Soluble	**Fructose** (as Fru, Glc/Fru, Hex or Suc/Fru)			

B. Harvest yield		Information included in models		
		Genotype, carbohydrates	Carbohydrates only	
		All genotype replicates	All genotype replicates	Averaged by genotype
Plants	Carbohydrate fractions	*R* values		
Mixed population^b^	All	0.79***	0.61***	0.81***
	Nonstructural	0.79***	0.62***	0.75***
	Soluble	0.79***	0.62***	0.75***
Mapping family 2013	All carbohydrates	0.85***	0.77***	0.70***
	Nonstructural	0.86***	0.65***	0.72***
	Soluble	0.86***	0.68***	0.72***
Mapping family 2014	Nonstructural	0.75***	0.56***	0.68***
	Soluble	0.74***	0.40***	0.58***
Carbohydrate fractions	Constituents common to predictors of all models^a^			
All	**Fructose** (as Fru, Glc/Fru or NSC)			
Nonstructural	**Fructose** (as Fru, Glc/Fru, NSC or Suc/Fru); **Glucose** (as Glc/Fru, NSC or Suc/Glc); **Sucrose** (as NSC, Suc/Fru, Suc/Glc or Suc/Sta); **Starch** (as NSC, Sta or Suc/Sta)			
Soluble	**Fructose** (as Fru, Glc/Fru or Suc/Fru)			

Support vector regression (*SMOreg*) models were trained using subsets of predictors selected (*CfsSubsetEval*) for individual correlation with trait but low correlation with each other. Models were evaluated in ninefold cross‐validations. *R* values indicate Pearson correlation between actual and predicted biomass data.

a Full lists in Supplementary Information.

b *M. sacchariflorus* genotype Mb306 was excluded from Table B.

As each experimental population comprised up to 102 genotypes, usually in biological replicates of only three, and distributed in field plots potentially subject to environmental gradients (Table S4), we were interested if genetic structure was detectable in the carbohydrate data. The effects on the *SMOreg* models of including or excluding genotype information for each replicate plant were therefore examined. Models of height (Table 2a) or harvest yield (Table 2b) were significantly improved (*P *<* *0.05, Wilcoxon tests) by appending genotype information to the carbohydrate predictors. Mean *R* of predicted vs. actual biomass values improved from 0.59 to 0.89 for height, and from 0.61 to 0.80 for yield, when models had prior knowledge of genotypes. This evidence for genetically conditioned ‘carbohydrate phenotypes’ was supported by the significantly better performance (*P *<* *0.05, Wilcoxon tests) of models constructed on averaged replicates of each genotype (mean *R* values: 0.73 for height; 0.71 for yield), relative to those based on all replicates (Table 2). This was presumably due to improved signal to noise for each genotype.

Models based on all carbohydrates including cell wall constituents, or on NSC, or on soluble carbohydrates, were all statistically significant (Table 2) and suggested that the more extensive analytical procedures were unlikely to prove essential in screening of *Miscanthus* populations for biomass potential. The numbers of predictors selected in the machine‐learning models ranged from 4 to 8 for the ‘all carbohydrates models’, 3 to 5 for the ‘NSC models’ and 2 to 4 for the ‘soluble carbohydrates models’, and detailed lists are available in Table S5. For each category of model, Table 2 highlights the carbohydrates that featured in every predictor list, whether as an individual metabolite or in a sum or ratio metric. The single metabolite that featured in every model in all categories was fructose. The other NSCs were prominent in some model categories, but ignored in others, with sucrose featuring the least frequently. Amongst cell wall constituents, glucan was ubiquitous in the all‐carbohydrate models of height (Table 2a), but not yield (Table 2b).

In modelling yield for the mixed population, one *M. sacchariflorus* genotype, Sac‐2, proved particularly detrimental (without genotype information) and was omitted for Table 2b. Amongst over four hundred plants sampled for this study, the two tallest individuals belonged to Sac‐2, but its yields per unit height were by far the lowest in the mixed population. Inclusion of this outlier genotype in the yield models saw *R* values for the mixed population (genotypes averaged) fall to 0.62, 0.36 and 0.52 for the all‐carbohydrate, NSC and soluble carbohydrate models, respectively. It was concluded that the traits responsible for the particular morphology of Sac‐2 were not accessible to modelling from carbohydrates.

**Figure 5:**
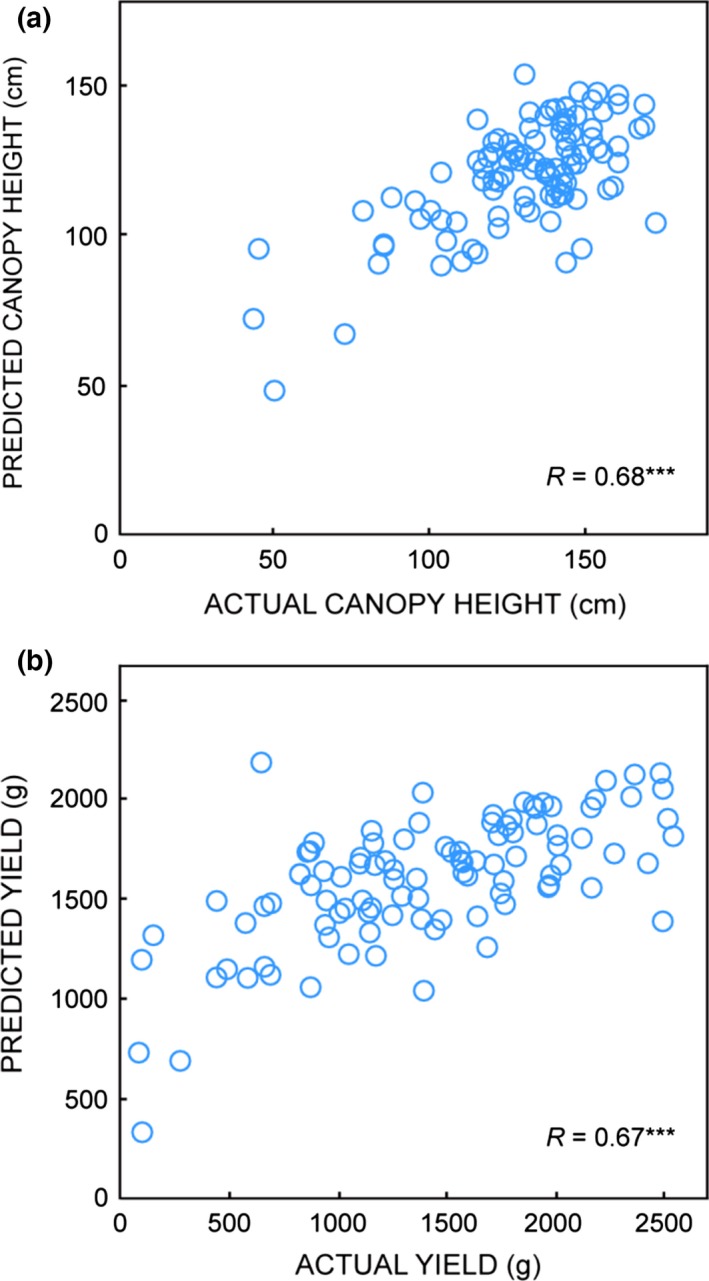
Interyear prediction of biomass traits from carbohydrates. From NSC levels in the 20 genotypes selected in 2013 from the mapping population, machine learning was used to select minimal lists of predictors to model biomass traits of the sampled plants. The machine‐learning models thereby ‘trained’ on the 2013 data were subsequently provided with the NSC data (only) of the 102 mapping family genotypes analysed in 2014 and ‘tested’ for prediction of (a) canopy height, and (b) yield, of the 2014 plants. For both models, the predictor metrics selected by machine learning were as follows: total NSC, and the Glc/Fru and Suc/Fru ratios. *R* indicates Pearson correlation (***, *P* < 0.001) between actual and predicted values.

Biomass and NSC data for the same genotypes in 2013 and 2014 showed high absolute (*R*) and rank (*R*_S_) interyear correlation (Table 3). We therefore investigated whether predictive models relating carbohydrates to biomass traits could be applicable from 1 year to another. Figure 5 shows the application of the machine‐learning regression method to prediction of (a) height, and (b) yield of the mapping family genotypes analysed in 2014, based purely on the measurements of their NSC levels (Table S6). The regression models were pretrained on the NSC and biomass data of the smaller number of mapping genotypes analysed in 2013. Correlations between predicted and actual biomass trait values were highly significant (*P *< 10^−14^) for height and yield, with Pearson correlation values of 0.67–0.68 (Fig. 5). Predictions for growth rate data were weaker (*R*, 0.25; *P *<* *0.05).

**Table 3:** Correlations between 2013 and 2014 for biomass traits and nonstructural carbohydrate composition. For biomass traits, *N *=* *102, and for carbohydrates and ratios, *N *=* *20, *P =≤ 0.05 **P= ≤0.01

Trait	Pearson's (*R*)	Spearman's Rank (*R*)	*P*
Canopy Height	0.8	0.8	**
Yield	0.9	0.9	**
Glucose	0.6	0.6	**
Fructose	0.8	0.7	**
Hexose	0.7	0.4	*
Sucrose	0.7	0.6	**
Starch	0.8	0.8	**
Total NSC	0.8	0.8	**
Suc/Sta	0.7	0.7	**
Glc/Fru	0.7	0.8	**
Sta/Fru	0.9	0.7	**

To investigate our hypothesis of a competitive relationship between starch and cellulose biosynthesis, we conducted a ^13^C‐labelling experiment in the field. We used a *M. sinensis* genotype (‘Goliath’), which was comparatively slow‐growing, a fast‐growing hybrid genotype phylogenetically similar to Hyb 2 of the mixed population (*M. x giganteus*), and a *M. sacchariflorus* genotype phylogenetically similar to Sac‐2. Stems were harvested 30 h after labelling. The slower growing genotype, Goliath, partitioned significantly more pulse‐derived ^13^C into starch than the fast‐growing hybrid and *M. sacchariflorus* (Table 4). When analysed by anova, there appeared to be no difference between the genotypes in % deposition into the cell wall even though the mean values were quite different. We considered that the analysis was being skewed by a large amount of variation between replicate in the *M. sacchariflorus* genotype (Table 4). Therefore, we also performed *t*‐tests between the three genotypes, which showed that *M. x giganteus* had deposited significantly more pulse‐derived ^13^C into the insoluble fraction which would be mainly comprised of cellulose. This approach was also applied on other measurements (such as % soluble), and no additional significant differences between genotypes were observed. The *M. sacchariflorus* genotype, whilst taller than *M. x giganteus*, had a slower growth rate at the time of labelling and was not statistically distinct from either of the other genotypes in its pulse‐derived ^13^C deposition. All three genotypes had accumulated the same total amount of pulse‐derived ^13^C. Therefore, the observed differences between the hybrid and Goliath were in carbon partitioning rather than capture (Table 4).

**Table 4:** Biomass traits and % ^13^C recovered from the stem 30 h after labelling in three genotypes. *N* = 3 ± SE. Different letters show significant differences between genotypes according to an anova and associated *t*‐test. Values in parentheses in the % insoluble column show the additional results of *t*‐tests between the three genotypes (*P* =≤ 0.05)

	Stem weight (g DW)	Stem height (cm)	Growth rate (cm day^−1^)	Pulse derived ^13^C (mg g^−1^ DW)	^13^C recovered in each fraction as a % of the total		
					Soluble	Starch	Insoluble
Hybrid	30.0 ± 3.5 a	241.0 ± 11.6 a	2.8 ± 0.4 a	2.0 ± 0.2 a	19.0 ± 2.7 a	18.2 ± 2.8 a	62.8 ± 0.8 a (a)
Goliath	20.0 ± 1.1 ab	180.3 ± 2.0 b	1.0 ± 0.5 b	2.2 ± 0.4 a	17.4 ± 2.5 a	38.2 ± 4.8 b	44.4 ± 3.2 a (b)
*M. sacchariflorus*	54.1 ± 10.7 b	274.3 ± 3.5 c	1.3 ± 0.3 ab	1.5 ± 0.2 a	39.0 ± 12.3 a	26.3 ± 1.5 a	34.8 ± 13.8 a (ab)

**Figure 6:**
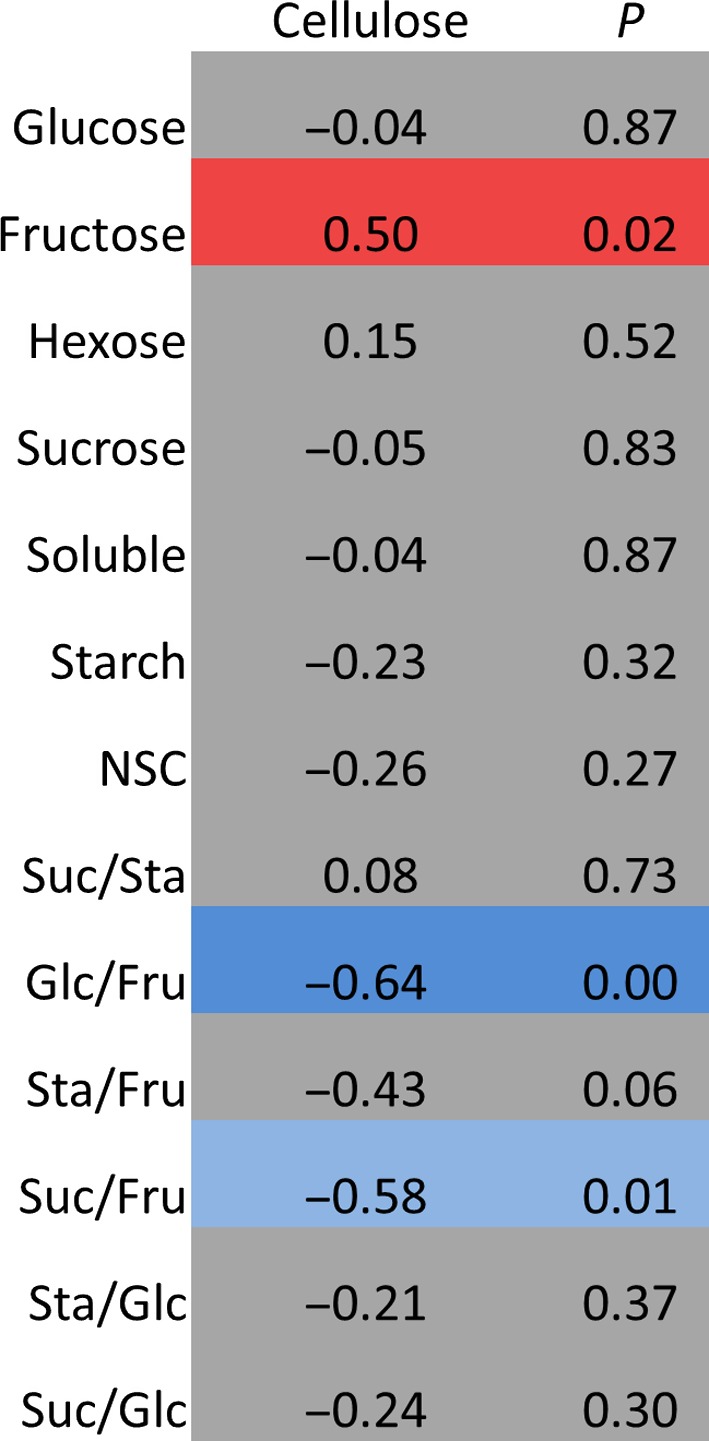
Spearman's rank correlation between cellulose and the NSC levels and ratios in the mapping family in 2013. Significant positive correlations are coloured red, and significant negative correlations are coloured blue (*P* =≤ 0.05). Nonsignificant correlations are coloured grey.

In the mapping family in 2013, there was a positive correlation between concentrations of cellulose and fructose, and negative relationships between cellulose and the ratios of glucose/fructose and sucrose/fructose (Fig. 6). However, no negative relationship between starch and cellulose was observed, which did not support our starting hypothesis that a competitive relationship exists between these two polymers of glucose (Fig. 6).

## Discussion

We have demonstrated that the abundance and partitioning of carbohydrates, particularly the NSC, can act as metabolic biomarkers of productivity in diverse genotypes of field‐grown *Miscanthus*. These findings thereby support other studies that have modelled biomass from metabolites using the model species *A. thaliana* in controlled environments (Meyer *et al*., 2007; Sulpice *et al*., 2009, 2010, 2013; Scott *et al*., 2010). The present article, by contrast, takes the important step of applying this approach to the field environment using a bioenergy crop species with a limited breeding history. Despite the likely complex impacts of sunlight, temperature and rainfall on the carbohydrate status of the field plants, informative and significant biomass models could be generated.

In our field‐grown Miscanthus, we observed that fructose consistently, positively correlated with yield traits. Fructose is produced exclusively from the metabolism of sucrose by the action of sucrose synthases (SuSy) and invertases, whereas glucose is produced both by the action of invertases (but not SuSy) and the metabolism of starch (Koch, 2004; Smith *et al*., 2005; Ruan, 2014). Therefore, fructose is a direct indication of sucrose metabolism, whereas glucose provides information about both sucrose and starch metabolism. Whilst our observations are strictly correlative (there is no evidence from our results that a high abundance of fructose specifically *causes* plants to be taller or higher yielding), there is evidence in the literature that altered partitioning in the ratio of sucrose/starch can follow increases in biomass in other species (Foyer & Ferrario, 1994; Laporte *et al*., 1997; Signora *et al*., 1998). For example, sucrose phosphate synthase (SPS) catalyses the penultimate step in the synthesis of sucrose in the cytosol (Signora *et al*., 1998). In tomato plants engineered to constitutively overexpress maize SPS, biomass was increased up to 100% compared to wild‐type controls (Foyer & Ferrario, 1994; Laporte *et al*., 1997). Furthermore, when the experiment was replicated in *Arabidopsis*, the ratio of sucrose to starch increased due to a reduction in starch in the transgenic plants (changes in biomass were not observed in this study; Signora *et al*., 1998). More recently, it has been found that transgenic expression of *Arabidopsis* SPS and sucrose phosphate phosphatase also enhanced growth and biomass accumulation in hybrid poplar (Maloney *et al*., 2015). Conversely, an *Arabidopsis* double knockout mutant of the major SPS leaf isoforms was strongly impaired in growth and accumulated high levels of starch (Volkert *et al*., 2014). In the Poaceae, SPS has been of interest as a biochemical marker for complex agronomic traits in several species (Castleden *et al*., 2004). In rice, an SPS gene coincides with a quantitative trait locus for plant height (Ishimaru *et al*., 2004; Venu *et al*., 2014), whilst plants with a maize SPS transgene grew taller (Ishimaru *et al*., 2004).

Sucrose‐metabolizing enzymes are candidates for explaining the correlation between hexose and biomass. The overexpression of SuSy and UDP‐glucose pyrophosphorylase (either as individual or as double mutants) in transgenic tobacco resulted in an increase in the abundance of hexoses, particularly fructose, a decrease in the glucose/fructose ratio and a concurrent increase in plant biomass (Coleman *et al*., 2006). Poovaiah *et al*. (Poovaiah *et al*., 2015) overexpressed a SuSy transgene in the biofuel feedstock switchgrass (*Panicum virgatum*), achieving increased height and biomass in some transformants. In *Populus alba* x *grandidentata* (hybrid poplar), overexpression of SuSy caused increases in soluble carbohydrates and cellulose, and a decrease in cell wall‐derived arabinose (Coleman *et al*., 2009). A plasma membrane‐bound isoform of SuSy is thought to transfer UDP‐glucose units directly to the extending glucan chains of cellulose (McFarlane *et al*., 2014), providing a link between the metabolism of the nonstructural pool and the formation of structural biomass. Some of these results therefore complement our findings using natural variation, in that the highest yielding plants had high hexoses, a low glucose/fructose ratio, higher cellulose and lower arabinose. Therefore, the abundance or activity of enzymes involved in sucrose biosynthesis or metabolism are candidates for the causal basis of our observations on hexoses and biomass.

No negative correlation between cellulose and starch was observed, which did not support our starting hypothesis that a competitive relationship exists. However, significant, negative correlations were observed between cellulose and the ratios of glucose/fructose and sucrose/fructose and a positive relationship between fructose and cellulose. This suggests that when the proportion of fructose is higher (relative to glucose or sucrose), cellulose is also in greater abundance. Cellulose biosynthesis is dependent on sucrose metabolism (Amor *et al*., 1995; Coleman *et al*., 2009; Baroja‐Fernandez *et al*., 2012), and the positive correlation between an increased proportion of fructose and cellulose could be a demonstration of this; as UDP‐glucose units, cleaved from sucrose through the action of SuSy, are transported across the plasma membrane to the extending cellulose chain, an increasing pool of fructose is left behind. Our findings from the ^13^C‐labelling experiment support this, as the differences in partitioning were observed between the fast‐ and slow‐growing genotype. Therefore, a more rapid rate of growth depends upon a greater accumulation of cellulose, via sucrose metabolism, rather than transient storage as starch.

The strength of the significant correlations found in our study is within the same range as those reported in pairwise analyses of molecular markers and traits. Correlations between short nucleotide polymorphisms (SNPs) and starch quality showed significant correlations (*R* ^*2*^) of 0.17–0.67 in rice. In a human asthma, study correlations between SNPs and lung physiology ranged from (*R* ^*2*^) ~0.3 to 0.9 (Kim & Xing, 2009; Kharabian‐Masouleh *et al*., 2012). This demonstrates that the strength of the correlation between the starch/fructose ratio (for example) of *R *=* *0.6–0.8 is within a range comparable to molecular markers. Height is the trait that best correlates with yield (*R* ^2^ = ~0.55; Robson *et al*., 2013). Using modelling to combine the strongest biomarkers, the predicted and actual yields of the mapping family in 2014 produced correlations of *R *=* *0.67 (*R* ^2^ = 0.44), which is a weaker predictor than height (Robson *et al*., 2013). However, whilst metabolic profiling was not a stronger predictor of final yield than height alone in *Miscanthus*, detailed knowledge of the relations of metabolism and biomass accumulation can be expected to yield powerful novel tools to accelerate and enhance energy plant breeding programmes (Robson *et al*., 2013). For example, in *Miscanthus,* the juvenile phase severely hinders early phenotypic selection (Robson *et al*., 2013), but if the metabolic profile could predict mature height in juvenile plants, metabolic biomarkers could then be used in a similar way to molecular markers. To address this hypothesis, the next stage of our experimentation is to screen first‐year seedlings and 1‐year‐old and 2‐year‐old plants to discover at what stage in development the glycome can be used as a biomarker for yield in mature plants. As *Miscanthus* takes 4 years from sowing seed to reach maturity, if the markers could only be used in the 2nd year of growth, this could reduce screening time by 50%. An alternative scenario in which yield prediction through biomarkers could be highly beneficial is in species such as trees where physical phenotyping is particularly challenging, or in screening for abiotic or biotic stress tolerances. It is also possible that metabolic and molecular markers could be used synergistically in breeding programmes to improve selection.

A concern about the use of metabolic biomarkers is their reliability, given that metabolites are dynamic and their absolute abundances will vary. However, a number of studies have demonstrated that the abundance of NSC and the ratios between different pools is under genetic control (Calenge *et al*., 2006; Purdy *et al*., 2015). Furthermore, it is generally accepted (and experimentally demonstrated, e.g. Table 3) that a high‐yielding genotype will consistently produce high yields compared to a low‐yielding type, even though climatic conditions over the course of a whole growing season may vary tremendously from year to year. As yield is implicitly dependent upon the NSC pool to form the structural biomass, it is logical that the NSC composition must also be genetically controlled and similar enough between years and within genotypes to produce consistent results in yield and quality traits. Many of the metabolites measured, such as glucose and most cell wall components, were found to be unreliable for predicting yield when used alone, as they only produced significant relationships with biomass in one or other of the field sites. In contrast, fructose, starch and several of the ratios were found to be consistent indicators of biomass traits in both sets of plants and in both years of study. Therefore, as with molecular markers, it is important to choose robust markers to produce reliable results.

The model generated for the mapping family was based on data from plants in their 3rd and 4th complete growing season in the field. Whilst this is considered a mature crop, it has been shown that yields continue to increase at least until the 5th year of growth (Robson *et al*., 2013). Therefore, the current model may underestimate yields in subsequent years and have to be reparameterized once the annual increase in yields has plateaued.

In conclusion, our study has shown that fructose and starch positively and negatively correlate with yield traits, respectively. The glycome in the summer growing season can be used as a biomarker to predict future harvest yields in the following year. Plants that partitioned a greater proportion of captured carbon into cellulose rather than starch attained greater biomass. Metabolic biomarker identification may also be an approach that could be adapted for other agronomic traits such as stress tolerance or disease resistance.

## Supporting information


**Table S1.** Biomass traits in the mixed population (a) and mapping family (b). The population consisted of *M. sinensis* (Sin), Hybrids (Hyb) and *M. sacchariflorus* (Sac) and the mapping family were all hybrids (*M. sacchariflorus* × *M. sinensis*) except a single *M. sinensis* genotype, Goliath. Statistics show differences between genotypes from anova (*P* =≤ 0.05). *N *=* *3, ±SE.

**Table S2.** NSC composition in the mixed population (a) and mapping family (b). All carbohydrates are in mg g^−1^ DW. Statistics show differences between all genotypes from anova (*P* =≤ 0.05). *N *=* *3, ±SE.

**Table S3.** Cell wall composition in a mixed population (a) and mapping family (b). All carbohydrates are in mg g^−1^ DW. Statistics show differences between all genotypes from anova (*P* =≤ 0.05). *N *=* *3, ±SE.

**Table S4.** Block effects across the two trials. For the mixed population *N *=* *18 and for the mapping family *N *=* *19. Statistics (*F* Pr) show the results of a one‐way anova with block as a treatment factor (Significant differences = ≤ 0.05).

**Table S5.** Carbohydrate predictors of biomass traits selected by *CfsSubsetEval* (in the Weka software) for each of the machine learning models in Table 2.

**Table S6.** NSC composition in the complete mapping family in 2014.

## References

[gcbb12418-bib-0001] Allison GG , Morris C , Clifton‐Brown J , Lister SJ , Donnison IS (2011) Genotypic variation in cell wall composition in a diverse set of 244 accessions of Miscanthus. Biomass and Bioenergy, 35, 4740–4747.

[gcbb12418-bib-0002] Amor Y , Haigler CH , Johnson S , Wainscott M , Delmer DP (1995) A membrane‐associated form of sucrose synthase and its potential role in synthesis of cellulose and callose in plants. Proceedings of the National Academy of Sciences of the United States of America, 92, 9353–9357. 7568131 10.1073/pnas.92.20.9353 PMC40983

[gcbb12418-bib-0004] Baroja‐Fernandez E , Munoz FJ , Li J *et al* (2012) Sucrose synthase activity in the sus1/sus2/sus3/sus4 Arabidopsis mutant is sufficient to support normal cellulose and starch production. Proceedings of the National Academy of Sciences of the United States of America, 109, 321–326. 22184213 10.1073/pnas.1117099109 PMC3252950

[gcbb12418-bib-0005] Biasi C , Pitkamaki AS , Tavi NM , Koponen HT , Martikainen PJ (2012) An isotope approach based on 13C pulse‐chase labelling vs. the root trenching method to separate heterotrophic and autotrophic respiration in cultivated peatlands. Boreal Environment Research, 17, 184–192.

[gcbb12418-bib-0006] Calenge F , Saliba‐Colombani V , Mahieu S , Loudet O , Daniel‐Vedele F , Krapp A (2006) Natural variation for carbohydrate content in Arabidopsis. Interaction with complex traits dissected by quantitative genetics. Plant Physiology, 141, 1630–1643. 16798941 10.1104/pp.106.082396 PMC1533913

[gcbb12418-bib-0007] Castleden CK , Aoki N , Gillespie VJ *et al* (2004) Evolution and function of the sucrose‐phosphate synthase gene families in wheat and other grasses. Plant Physiology, 135, 1753–1764. 15247374 10.1104/pp.104.042457 PMC519087

[gcbb12418-bib-0008] Coleman HD , Ellis DD , Gilbert M , Mansfield SD (2006) Up‐regulation of sucrose synthase and UDP‐glucose pyrophosphorylase impacts plant growth and metabolism. Plant Biotechnology Journal, 4, 87–101. 17177788 10.1111/j.1467-7652.2005.00160.x

[gcbb12418-bib-0009] Coleman HD , Yan J , Mansfield SD (2009) Sucrose synthase affects carbon partitioning to increase cellulose production and altered cell wall ultrastructure. Proceedings of the National Academy of Sciences of the United States of America, 106, 13118–13123. 19625620 10.1073/pnas.0900188106 PMC2722352

[gcbb12418-bib-0010] Foster CE , Martin TM , Pauly M (2010a) Comprehensive compositional analysis of plant cell walls (Lignocellulosic biomass) part I: lignin. Journal of Visualized Experiments, 1745, doi: 10.3791/1745. 10.3791/1745 PMC3144576 20224547

[gcbb12418-bib-0011] Foster CE , Martin TM , Pauly M (2010b) Comprehensive compositional analysis of plant cell walls (lignocellulosic biomass) part II: carbohydrates. Journal of Visualized Experiments, 1837, doi: 10.3791/1837. 10.3791/1837 PMC3145335 20228730

[gcbb12418-bib-0012] Foyer CH , Ferrario S (1994) Modulation of carbon and nitrogen‐metabolism in transgenic plants with a view to improved biomass production. Biochemical Society Transactions, 22, 909–915. 7698483 10.1042/bst0220909

[gcbb12418-bib-0013] Frank E , Hall M , Trigg L , Holmes G , Witten IH (2004) Data mining in bioinformatics using Weka. Bioinformatics, 20, 2479–2481. 15073010 10.1093/bioinformatics/bth261

[gcbb12418-bib-0014] Hodkinson TR , Chase MW , Lledo MD , Salamin N , Renvoize SA (2002) Phylogenetics of Miscanthus, Saccharum and related genera (Saccharinae, Andropogoneae, Poaceae) based on DNA sequences from ITS nuclear ribosomal DNA and plastid trnL intron and trnL‐F intergenic spacers. Journal of Plant Research, 115, 381–392. 12579363 10.1007/s10265-002-0049-3

[gcbb12418-bib-0015] Hogberg P , Hogberg MN , Gottlicher SG *et al* (2008) High temporal resolution tracing of photosynthate carbon from the tree canopy to forest soil microorganisms. New Phytologist, 177, 220–228. 17944822 10.1111/j.1469-8137.2007.02238.x

[gcbb12418-bib-0016] Ishimaru K , Ono K , Kashiwagi T (2004) Identification of a new gene controlling plant height in rice using the candidate‐gene strategy. Planta, 218, 388–395. 14534788 10.1007/s00425-003-1119-z

[gcbb12418-bib-0017] Jensen E , Farrar K , Thomas‐Jones S , Hastings A , Donnison I , Clifton‐Brown J (2011) Characterization of flowering time diversity in Miscanthus species. GCB Bioenergy, 3, 387–400.

[gcbb12418-bib-0018] Jones MG , Outlaw WH , Lowry OH (1977) Enzymic assay of 10 to 10 moles of sucrose in plant tissues. Plant Physiology, 60, 379–383. 16660097 10.1104/pp.60.3.379 PMC542620

[gcbb12418-bib-0019] Kharabian‐Masouleh A , Waters DLE , Reinke RF , Ward R , Henry RJ (2012) SNP in starch biosynthesis genes associated with nutritional and functional properties of rice. Scientific Reports, 2, 557. 22870386 10.1038/srep00557 PMC3412280

[gcbb12418-bib-0020] Kim S , Xing EP (2009) Statistical estimation of correlated genome associations to a quantitative trait network. Plos Genetics, 5, e1000587. 19680538 10.1371/journal.pgen.1000587 PMC2719086

[gcbb12418-bib-0021] Koch K (2004) Sucrose metabolism: regulatory mechanisms and pivotal roles in sugar sensing and plant development. Current Opinion in Plant Biology, 7, 235–246. 15134743 10.1016/j.pbi.2004.03.014

[gcbb12418-bib-0022] Laporte MM , Galagan JA , Shapiro JA , Boersig MR , Shewmaker CK , Sharkey TD (1997) Sucrose‐phosphate synthase activity and yield analysis of tomato plants transformed with maize sucrose‐phosphate synthase. Planta, 203, 253–259. 10.1007/s004250000433 11346956

[gcbb12418-bib-0023] Li HD , Liang YZ , Xu QS (2009) Support vector machines and its applications in chemistry. Chemometrics and Intelligent Laboratory Systems, 95, 188–198.

[gcbb12418-bib-0024] Maloney VJ , Park JY , Unda F , Mansfield SD (2015) Sucrose phosphate synthase and sucrose phosphate phosphatase interact in planta and promote plant growth and biomass accumulation. Journal of Experimental Botany, 66, 4383–4394. 25873678 10.1093/jxb/erv101 PMC4493782

[gcbb12418-bib-0025] McFarlane HE , Doring A , Persson S (2014) The cell biology of cellulose synthesis. Annual Review of Plant Biology, 65, 69. 10.1146/annurev-arplant-050213-040240 24579997

[gcbb12418-bib-0026] Meyer RC , Steinfath M , Lisec J *et al* (2007) The metabolic signature related to high plant growth rate in *Arabidopsis thaliana* . Proceedings of the National Academy of Sciences of the United States of America, 104, 4759–4764. 17360597 10.1073/pnas.0609709104 PMC1810331

[gcbb12418-bib-0027] Muguerza M , Gondo T , Yoshida M , Kawakami A , Terami F , Yamada T , Akashi R (2013) Modification of the total soluble sugar content of the C4 grass *Paspalum notatum* expressing the wheat‐derived sucrose: sucrose 1‐fructosyltransferase and sucrose: fructan 6‐fructosyltransferase genes. Grassland Science, 59, 196–204.

[gcbb12418-bib-0028] O'Brien MJ , Leuzinger S , Philipson CD , Tay J , Hector A (2014) Drought survival of tropical tree seedlings enhanced by non‐structural carbohydrate levels. Nature Climate Change, 4, 710–714.

[gcbb12418-bib-0029] Poovaiah CR , Mazarei M , Decker SR , Turner GB , Sykes RW , Davis MF , Stewart CN Jr (2015) Transgenic switchgrass (*Panicum virgatum* L.) biomass is increased by overexpression of switchgrass sucrose synthase (PvSUS1). Biotechnology Journal, 10, 552–563. 25327983 10.1002/biot.201400499

[gcbb12418-bib-0030] Purdy SJ , Maddison AL , Jones LE , Webster RJ , Andralojc J , Donnison I , Clifton‐Brown J (2013) Characterization of chilling‐shock responses in four genotypes of *Miscanthus* reveals the superior tolerance of *M. x giganteus* compared with *M. sinensis* and *M. sacchariflorus* . Annals of Botany, 111, 999–1013. 23519835 10.1093/aob/mct059 PMC3631343

[gcbb12418-bib-0031] Purdy SJ , Cunniff J , Maddison AL *et al* (2014) Seasonal carbohydrate dynamics and climatic regulation of senescence in the perennial grass, Miscanthus. Bioenergy Research, 8, 28–41.

[gcbb12418-bib-0032] Purdy SJ , Maddison AL , Cunniff J , Donnison I , Clifton‐Brown J (2015) Non‐structural carbohydrate profiles and ratios between soluble sugars and starch serve as indicators of productivity for a bioenergy grass. AoB Plants, 7, plv032, doi: 10.1093/aobpla/plv032. 10.1093/aobpla/plv032 PMC5024741 25829378

[gcbb12418-bib-0033] Robson P , Mos M , Clifton‐Brown J , Donnison I (2012) Phenotypic variation in senescence in Miscanthus: towards optimising biomass quality and quantity. Bioenergy Research, 5, 95–105.

[gcbb12418-bib-0034] Robson P , Jensen E , Hawkins S *et al* (2013) Accelerating the domestication of a bioenergy crop: identifying and modelling morphological targets for sustainable yield increase in Miscanthus. Journal of Experimental Botany, 64, 4143–4155. 24064927 10.1093/jxb/ert225 PMC3808307

[gcbb12418-bib-0035] Rocher JP (1988) Comparison of carbohydrate compartmentation in relation to photosynthesis, assimilate export and growth in a range of maize genotypes. Australian Journal of Plant Physiology, 15, 677–686.

[gcbb12418-bib-0036] Ruan YL (2014) Sucrose metabolism: gateway to diverse carbon use and sugar signaling. Annual Review of Plant Biology, Vol 61, 65, 33–67. 10.1146/annurev-arplant-050213-040251 24579990

[gcbb12418-bib-0037] Scott IM , Vermeer CP , Liakata M *et al* (2010) Enhancement of plant metabolite fingerprinting by machine learning. Plant Physiology, 153, 1506–1520. 20566707 10.1104/pp.109.150524 PMC2923910

[gcbb12418-bib-0038] Scott IM , Ward JL , Miller SJ , Beale MH (2014) Opposite variations in fumarate and malate dominate metabolic phenotypes of Arabidopsis salicylate mutants with abnormal biomass under chilling. Physiologia Plantarum, 152, 660–674. 24735077 10.1111/ppl.12210

[gcbb12418-bib-0039] Signora L , Galtier N , Skot L , Lucas H , Foyer CH (1998) Over‐expression of sucrose phosphate synthase in *Arabidopsis thaliana* results in increased foliar sucrose/starch ratios and favours decreased foliar carbohydrate accumulation in plants after prolonged growth with CO_2_ enrichment. Journal of Experimental Botany, 49, 669–680.

[gcbb12418-bib-0040] Smith AM , Zeeman SC , Smith SM (2005) Starch degradation. Annual Review of Plant Biology, 56, 73–98. 10.1146/annurev.arplant.56.032604.144257 15862090

[gcbb12418-bib-0041] Somerville C , Youngs H , Taylor C , Davis SC , Long SP (2010) Feedstocks for Lignocellulosic Biofuels. Science, 329, 790–792. 20705851 10.1126/science.1189268

[gcbb12418-bib-0042] de Souza AP , Arundale RA , Dohleman FG , Long SP , Buckeridge MS (2013) Will the exceptional productivity of Miscanthus x giganteus increase further under rising atmospheric CO_2_? Agricultural and Forest Meteorology, 171, 82–92.

[gcbb12418-bib-0043] Steinfath M , Strehmel N , Peters R *et al* (2010) Discovering plant metabolic biomarkers for phenotype prediction using an untargeted approach. Plant Biotechnology Journal, 8, 900–911. 20353402 10.1111/j.1467-7652.2010.00516.x

[gcbb12418-bib-0044] Subke JA , Vallack HW , Magnusson T , Keel SG , Metcalfe DB , Hogberg P , Ineson P (2009) Short‐term dynamics of abiotic and biotic soil ^13^CO_2_ effluxes after *in situ* ^13^CO_2_ pulse labelling of a boreal pine forest. New Phytologist, 183, 349–357. 19496953 10.1111/j.1469-8137.2009.02883.x

[gcbb12418-bib-0045] Sulpice R , Pyl ET , Ishihara H *et al* (2009) Starch as a major integrator in the regulation of plant growth. Proceedings of the National Academy of Sciences of the United States of America, 106, 10348–10353. 19506259 10.1073/pnas.0903478106 PMC2693182

[gcbb12418-bib-0046] Sulpice R , Trenkamp S , Steinfath M *et al* (2010) Network analysis of enzyme activities and metabolite levels and their relationship to biomass in a large panel of Arabidopsis accessions. Plant Cell, 22, 2872–2893. 20699391 10.1105/tpc.110.076653 PMC2947169

[gcbb12418-bib-0047] Sulpice R , Nikoloski Z , Tschoep H *et al* (2013) Impact of the carbon and nitrogen supply on relationships and connectivity between metabolism and biomass in a broad panel of Arabidopsis accessions. Plant Physiology, 162, 347–363. 23515278 10.1104/pp.112.210104 PMC3641214

[gcbb12418-bib-0048] Venu RC , Ma J , Jia Y *et al* (2014) Identification of candidate genes associated with positive and negative heterosis in rice. PLoS ONE, 9, e95178. 24743656 10.1371/journal.pone.0095178 PMC3990613

[gcbb12418-bib-0049] Visser P , Pignatelli V (2001) Utilization of Miscanthus In: Miscanthus: For Energy and Fibre (ed. Walsh M), pp. 109–154. Earthscan, London.

[gcbb12418-bib-0050] Volkert K , Debast S , Voll LM *et al* (2014) Loss of the two major leaf isoforms of sucrose‐phosphate synthase in *Arabidopsis thaliana* limits sucrose synthesis and nocturnal starch degradation but does not alter carbon partitioning during photosynthesis. Journal of Experimental Botany, 65, 5217–5229. 24994761 10.1093/jxb/eru282 PMC4400537

[gcbb12418-bib-0051] Wang L , Wu JQ (2013) The essential role of Jasmonic acid in plant‐herbivore interactions – using the wild tobacco *Nicotiana attenuata* as a model. Journal of Genetics and Genomics, 40, 597–606. 24377866 10.1016/j.jgg.2013.10.001

[gcbb12418-bib-0052] Wang Y , Tetko IV , Hall MA , Frank E , Facius A , Mayer KFX , Mewes HW (2005) Gene selection from microarray data for cancer classification – a machine learning approach. Computational Biology and Chemistry, 29, 37–46. 15680584 10.1016/j.compbiolchem.2004.11.001

[gcbb12418-bib-0053] Wanner LA , Junttila O (1999) Cold‐induced freezing tolerance in Arabidopsis. Plant Physiology, 120, 391–399. 10364390 10.1104/pp.120.2.391 PMC59277

