# Supplementary material for: Predicting future biomass yield in Miscanthus using the carbohydrate metabolic profile as a biomarker
Source: Glob Change Biol Bioenergy. 2017 Jan 21;9(7):1264–78. doi: 10.1111/gcbb.12418 (PMC5488626; doi:10.1111/gcbb.12418)
Supplement: Supplementary file 1 — Table S1. Biomass traits in the mixed population (a) and mapping family (b). The population consisted of M. sinensis (Sin), Hybrids (Hyb) and M. sacchariflorus (Sac) and the mapping family were all hybrids (M. sacchariflorus × M. sinensis) except a single M. sinensis genotype, Goliath. Statistics show differences between genotypes from anova (P =≤ 0.05). N = 3, ±SE. [file GCBB-9-1264-s001.pptx]

## Slide 1
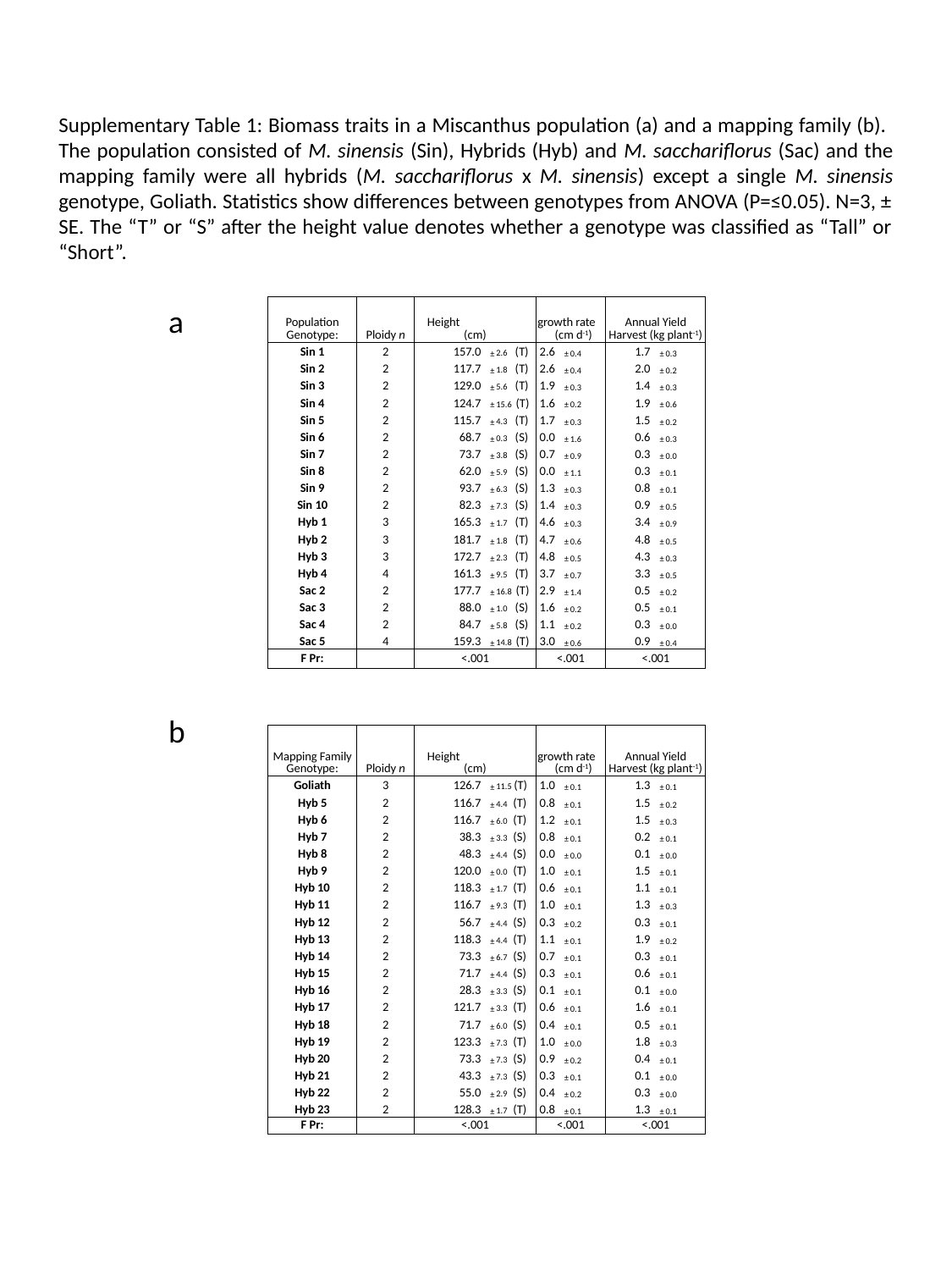

Supplementary Table 1: Biomass traits in a Miscanthus population (a) and a mapping family (b). The population consisted of M. sinensis (Sin), Hybrids (Hyb) and M. sacchariflorus (Sac) and the mapping family were all hybrids (M. sacchariflorus x M. sinensis) except a single M. sinensis genotype, Goliath. Statistics show differences between genotypes from ANOVA (P=≤0.05). N=3, ± SE. The “T” or “S” after the height value denotes whether a genotype was classified as “Tall” or “Short”.
a
| Population Genotype: | Ploidy n | Height (cm) | | | growth rate (cm d-1) | | | Annual Yield Harvest (kg plant-1) | | |
| --- | --- | --- | --- | --- | --- | --- | --- | --- | --- | --- |
| Sin 1 | 2 | 157.0 | ± | 2.6 (T) | 2.6 | ± | 0.4 | 1.7 | ± | 0.3 |
| Sin 2 | 2 | 117.7 | ± | 1.8 (T) | 2.6 | ± | 0.4 | 2.0 | ± | 0.2 |
| Sin 3 | 2 | 129.0 | ± | 5.6 (T) | 1.9 | ± | 0.3 | 1.4 | ± | 0.3 |
| Sin 4 | 2 | 124.7 | ± | 15.6 (T) | 1.6 | ± | 0.2 | 1.9 | ± | 0.6 |
| Sin 5 | 2 | 115.7 | ± | 4.3 (T) | 1.7 | ± | 0.3 | 1.5 | ± | 0.2 |
| Sin 6 | 2 | 68.7 | ± | 0.3 (S) | 0.0 | ± | 1.6 | 0.6 | ± | 0.3 |
| Sin 7 | 2 | 73.7 | ± | 3.8 (S) | 0.7 | ± | 0.9 | 0.3 | ± | 0.0 |
| Sin 8 | 2 | 62.0 | ± | 5.9 (S) | 0.0 | ± | 1.1 | 0.3 | ± | 0.1 |
| Sin 9 | 2 | 93.7 | ± | 6.3 (S) | 1.3 | ± | 0.3 | 0.8 | ± | 0.1 |
| Sin 10 | 2 | 82.3 | ± | 7.3 (S) | 1.4 | ± | 0.3 | 0.9 | ± | 0.5 |
| Hyb 1 | 3 | 165.3 | ± | 1.7 (T) | 4.6 | ± | 0.3 | 3.4 | ± | 0.9 |
| Hyb 2 | 3 | 181.7 | ± | 1.8 (T) | 4.7 | ± | 0.6 | 4.8 | ± | 0.5 |
| Hyb 3 | 3 | 172.7 | ± | 2.3 (T) | 4.8 | ± | 0.5 | 4.3 | ± | 0.3 |
| Hyb 4 | 4 | 161.3 | ± | 9.5 (T) | 3.7 | ± | 0.7 | 3.3 | ± | 0.5 |
| Sac 2 | 2 | 177.7 | ± | 16.8 (T) | 2.9 | ± | 1.4 | 0.5 | ± | 0.2 |
| Sac 3 | 2 | 88.0 | ± | 1.0 (S) | 1.6 | ± | 0.2 | 0.5 | ± | 0.1 |
| Sac 4 | 2 | 84.7 | ± | 5.8 (S) | 1.1 | ± | 0.2 | 0.3 | ± | 0.0 |
| Sac 5 | 4 | 159.3 | ± | 14.8 (T) | 3.0 | ± | 0.6 | 0.9 | ± | 0.4 |
| F Pr: | | <.001 | | | <.001 | | | <.001 | | |
| | | | | | | | | | | |
| | | | | | | | | | | |
| Mapping Family Genotype: | Ploidy n | Height (cm) | | | growth rate (cm d-1) | | | Annual Yield Harvest (kg plant-1) | | |
| Goliath | 3 | 126.7 | ± | 11.5 (T) | 1.0 | ± | 0.1 | 1.3 | ± | 0.1 |
| Hyb 5 | 2 | 116.7 | ± | 4.4 (T) | 0.8 | ± | 0.1 | 1.5 | ± | 0.2 |
| Hyb 6 | 2 | 116.7 | ± | 6.0 (T) | 1.2 | ± | 0.1 | 1.5 | ± | 0.3 |
| Hyb 7 | 2 | 38.3 | ± | 3.3 (S) | 0.8 | ± | 0.1 | 0.2 | ± | 0.1 |
| Hyb 8 | 2 | 48.3 | ± | 4.4 (S) | 0.0 | ± | 0.0 | 0.1 | ± | 0.0 |
| Hyb 9 | 2 | 120.0 | ± | 0.0 (T) | 1.0 | ± | 0.1 | 1.5 | ± | 0.1 |
| Hyb 10 | 2 | 118.3 | ± | 1.7 (T) | 0.6 | ± | 0.1 | 1.1 | ± | 0.1 |
| Hyb 11 | 2 | 116.7 | ± | 9.3 (T) | 1.0 | ± | 0.1 | 1.3 | ± | 0.3 |
| Hyb 12 | 2 | 56.7 | ± | 4.4 (S) | 0.3 | ± | 0.2 | 0.3 | ± | 0.1 |
| Hyb 13 | 2 | 118.3 | ± | 4.4 (T) | 1.1 | ± | 0.1 | 1.9 | ± | 0.2 |
| Hyb 14 | 2 | 73.3 | ± | 6.7 (S) | 0.7 | ± | 0.1 | 0.3 | ± | 0.1 |
| Hyb 15 | 2 | 71.7 | ± | 4.4 (S) | 0.3 | ± | 0.1 | 0.6 | ± | 0.1 |
| Hyb 16 | 2 | 28.3 | ± | 3.3 (S) | 0.1 | ± | 0.1 | 0.1 | ± | 0.0 |
| Hyb 17 | 2 | 121.7 | ± | 3.3 (T) | 0.6 | ± | 0.1 | 1.6 | ± | 0.1 |
| Hyb 18 | 2 | 71.7 | ± | 6.0 (S) | 0.4 | ± | 0.1 | 0.5 | ± | 0.1 |
| Hyb 19 | 2 | 123.3 | ± | 7.3 (T) | 1.0 | ± | 0.0 | 1.8 | ± | 0.3 |
| Hyb 20 | 2 | 73.3 | ± | 7.3 (S) | 0.9 | ± | 0.2 | 0.4 | ± | 0.1 |
| Hyb 21 | 2 | 43.3 | ± | 7.3 (S) | 0.3 | ± | 0.1 | 0.1 | ± | 0.0 |
| Hyb 22 | 2 | 55.0 | ± | 2.9 (S) | 0.4 | ± | 0.2 | 0.3 | ± | 0.0 |
| Hyb 23 | 2 | 128.3 | ± | 1.7 (T) | 0.8 | ± | 0.1 | 1.3 | ± | 0.1 |
| F Pr: | | <.001 | | | <.001 | | | <.001 | | |
b
