# Supplementary material for: Predicting future biomass yield in Miscanthus using the carbohydrate metabolic profile as a biomarker
Source: Glob Change Biol Bioenergy. 2017 Jan 21;9(7):1264–78. doi: 10.1111/gcbb.12418 (PMC5488626; doi:10.1111/gcbb.12418)
Supplement: Supplementary file 2 — Table S2. NSC composition in the mixed population (a) and mapping family (b). All carbohydrates are in mg g−1 DW. Statistics show differences between all genotypes from anova (P =≤ 0.05). N = 3, ±SE. [file GCBB-9-1264-s002.pptx]

## Slide 1
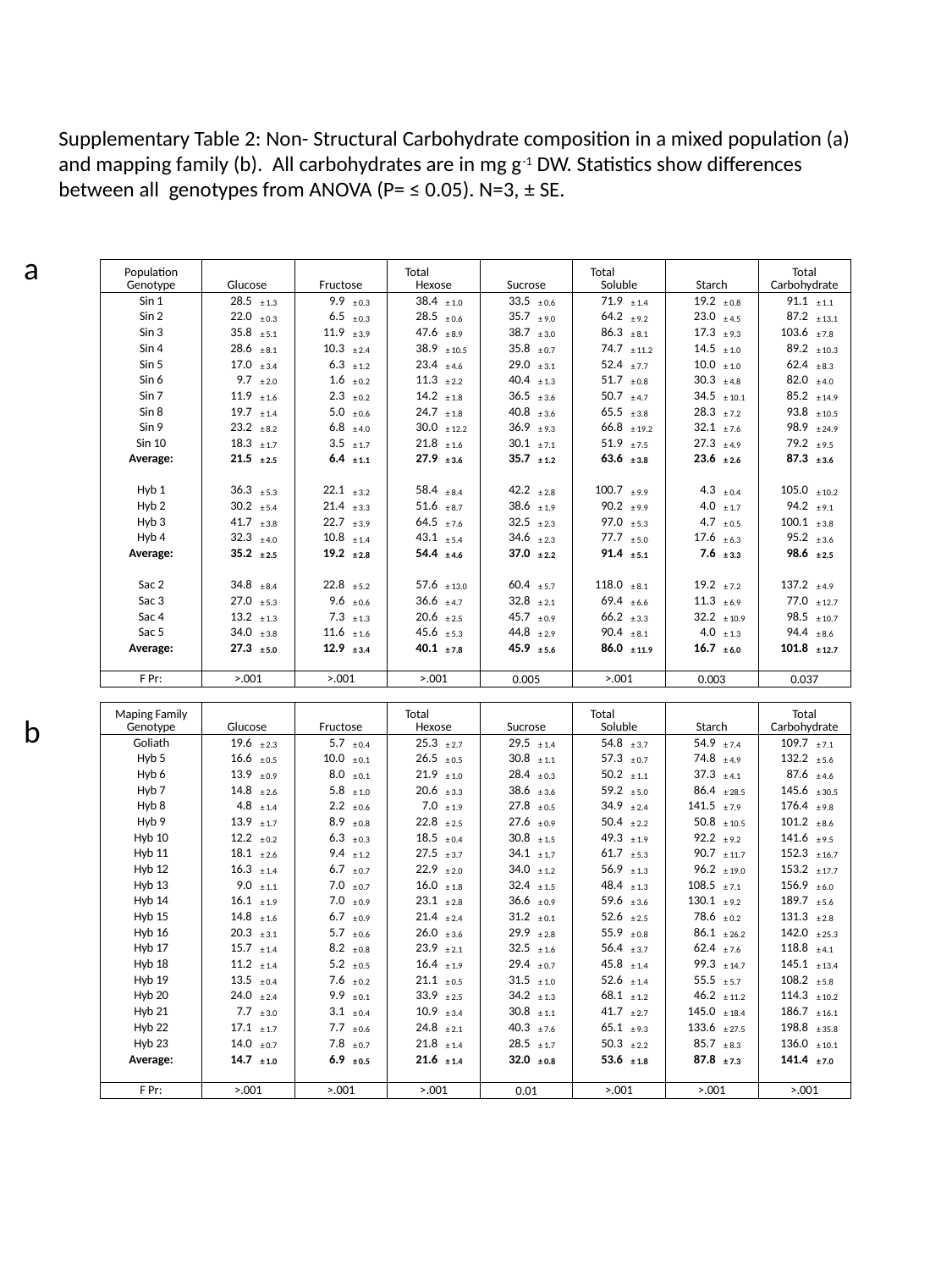

Supplementary Table 2: Non- Structural Carbohydrate composition in a mixed population (a) and mapping family (b). All carbohydrates are in mg g-1 DW. Statistics show differences between all genotypes from ANOVA (P= ≤ 0.05). N=3, ± SE.
a
| Population Genotype | Glucose | | | Fructose | | | Total Hexose | | | Sucrose | | | Total Soluble | | | Starch | | | Total Carbohydrate | | |
| --- | --- | --- | --- | --- | --- | --- | --- | --- | --- | --- | --- | --- | --- | --- | --- | --- | --- | --- | --- | --- | --- |
| Sin 1 | 28.5 | ± | 1.3 | 9.9 | ± | 0.3 | 38.4 | ± | 1.0 | 33.5 | ± | 0.6 | 71.9 | ± | 1.4 | 19.2 | ± | 0.8 | 91.1 | ± | 1.1 |
| Sin 2 | 22.0 | ± | 0.3 | 6.5 | ± | 0.3 | 28.5 | ± | 0.6 | 35.7 | ± | 9.0 | 64.2 | ± | 9.2 | 23.0 | ± | 4.5 | 87.2 | ± | 13.1 |
| Sin 3 | 35.8 | ± | 5.1 | 11.9 | ± | 3.9 | 47.6 | ± | 8.9 | 38.7 | ± | 3.0 | 86.3 | ± | 8.1 | 17.3 | ± | 9.3 | 103.6 | ± | 7.8 |
| Sin 4 | 28.6 | ± | 8.1 | 10.3 | ± | 2.4 | 38.9 | ± | 10.5 | 35.8 | ± | 0.7 | 74.7 | ± | 11.2 | 14.5 | ± | 1.0 | 89.2 | ± | 10.3 |
| Sin 5 | 17.0 | ± | 3.4 | 6.3 | ± | 1.2 | 23.4 | ± | 4.6 | 29.0 | ± | 3.1 | 52.4 | ± | 7.7 | 10.0 | ± | 1.0 | 62.4 | ± | 8.3 |
| Sin 6 | 9.7 | ± | 2.0 | 1.6 | ± | 0.2 | 11.3 | ± | 2.2 | 40.4 | ± | 1.3 | 51.7 | ± | 0.8 | 30.3 | ± | 4.8 | 82.0 | ± | 4.0 |
| Sin 7 | 11.9 | ± | 1.6 | 2.3 | ± | 0.2 | 14.2 | ± | 1.8 | 36.5 | ± | 3.6 | 50.7 | ± | 4.7 | 34.5 | ± | 10.1 | 85.2 | ± | 14.9 |
| Sin 8 | 19.7 | ± | 1.4 | 5.0 | ± | 0.6 | 24.7 | ± | 1.8 | 40.8 | ± | 3.6 | 65.5 | ± | 3.8 | 28.3 | ± | 7.2 | 93.8 | ± | 10.5 |
| Sin 9 | 23.2 | ± | 8.2 | 6.8 | ± | 4.0 | 30.0 | ± | 12.2 | 36.9 | ± | 9.3 | 66.8 | ± | 19.2 | 32.1 | ± | 7.6 | 98.9 | ± | 24.9 |
| Sin 10 | 18.3 | ± | 1.7 | 3.5 | ± | 1.7 | 21.8 | ± | 1.6 | 30.1 | ± | 7.1 | 51.9 | ± | 7.5 | 27.3 | ± | 4.9 | 79.2 | ± | 9.5 |
| Average: | 21.5 | ± | 2.5 | 6.4 | ± | 1.1 | 27.9 | ± | 3.6 | 35.7 | ± | 1.2 | 63.6 | ± | 3.8 | 23.6 | ± | 2.6 | 87.3 | ± | 3.6 |
| | | | | | | | | | | | | | | | | | | | | | |
| Hyb 1 | 36.3 | ± | 5.3 | 22.1 | ± | 3.2 | 58.4 | ± | 8.4 | 42.2 | ± | 2.8 | 100.7 | ± | 9.9 | 4.3 | ± | 0.4 | 105.0 | ± | 10.2 |
| Hyb 2 | 30.2 | ± | 5.4 | 21.4 | ± | 3.3 | 51.6 | ± | 8.7 | 38.6 | ± | 1.9 | 90.2 | ± | 9.9 | 4.0 | ± | 1.7 | 94.2 | ± | 9.1 |
| Hyb 3 | 41.7 | ± | 3.8 | 22.7 | ± | 3.9 | 64.5 | ± | 7.6 | 32.5 | ± | 2.3 | 97.0 | ± | 5.3 | 4.7 | ± | 0.5 | 100.1 | ± | 3.8 |
| Hyb 4 | 32.3 | ± | 4.0 | 10.8 | ± | 1.4 | 43.1 | ± | 5.4 | 34.6 | ± | 2.3 | 77.7 | ± | 5.0 | 17.6 | ± | 6.3 | 95.2 | ± | 3.6 |
| Average: | 35.2 | ± | 2.5 | 19.2 | ± | 2.8 | 54.4 | ± | 4.6 | 37.0 | ± | 2.2 | 91.4 | ± | 5.1 | 7.6 | ± | 3.3 | 98.6 | ± | 2.5 |
| | | | | | | | | | | | | | | | | | | | | | |
| Sac 2 | 34.8 | ± | 8.4 | 22.8 | ± | 5.2 | 57.6 | ± | 13.0 | 60.4 | ± | 5.7 | 118.0 | ± | 8.1 | 19.2 | ± | 7.2 | 137.2 | ± | 4.9 |
| Sac 3 | 27.0 | ± | 5.3 | 9.6 | ± | 0.6 | 36.6 | ± | 4.7 | 32.8 | ± | 2.1 | 69.4 | ± | 6.6 | 11.3 | ± | 6.9 | 77.0 | ± | 12.7 |
| Sac 4 | 13.2 | ± | 1.3 | 7.3 | ± | 1.3 | 20.6 | ± | 2.5 | 45.7 | ± | 0.9 | 66.2 | ± | 3.3 | 32.2 | ± | 10.9 | 98.5 | ± | 10.7 |
| Sac 5 | 34.0 | ± | 3.8 | 11.6 | ± | 1.6 | 45.6 | ± | 5.3 | 44.8 | ± | 2.9 | 90.4 | ± | 8.1 | 4.0 | ± | 1.3 | 94.4 | ± | 8.6 |
| Average: | 27.3 | ± | 5.0 | 12.9 | ± | 3.4 | 40.1 | ± | 7.8 | 45.9 | ± | 5.6 | 86.0 | ± | 11.9 | 16.7 | ± | 6.0 | 101.8 | ± | 12.7 |
| | | | | | | | | | | | | | | | | | | | | | |
| F Pr: | >.001 | | | >.001 | | | >.001 | | | 0.005 | | | >.001 | | | 0.003 | | | 0.037 | | |
| | | | | | | | | | | | | | | | | | | | | | |
| Maping Family Genotype | Glucose | | | Fructose | | | Total Hexose | | | Sucrose | | | Total Soluble | | | Starch | | | Total Carbohydrate | | |
| Goliath | 19.6 | ± | 2.3 | 5.7 | ± | 0.4 | 25.3 | ± | 2.7 | 29.5 | ± | 1.4 | 54.8 | ± | 3.7 | 54.9 | ± | 7.4 | 109.7 | ± | 7.1 |
| Hyb 5 | 16.6 | ± | 0.5 | 10.0 | ± | 0.1 | 26.5 | ± | 0.5 | 30.8 | ± | 1.1 | 57.3 | ± | 0.7 | 74.8 | ± | 4.9 | 132.2 | ± | 5.6 |
| Hyb 6 | 13.9 | ± | 0.9 | 8.0 | ± | 0.1 | 21.9 | ± | 1.0 | 28.4 | ± | 0.3 | 50.2 | ± | 1.1 | 37.3 | ± | 4.1 | 87.6 | ± | 4.6 |
| Hyb 7 | 14.8 | ± | 2.6 | 5.8 | ± | 1.0 | 20.6 | ± | 3.3 | 38.6 | ± | 3.6 | 59.2 | ± | 5.0 | 86.4 | ± | 28.5 | 145.6 | ± | 30.5 |
| Hyb 8 | 4.8 | ± | 1.4 | 2.2 | ± | 0.6 | 7.0 | ± | 1.9 | 27.8 | ± | 0.5 | 34.9 | ± | 2.4 | 141.5 | ± | 7.9 | 176.4 | ± | 9.8 |
| Hyb 9 | 13.9 | ± | 1.7 | 8.9 | ± | 0.8 | 22.8 | ± | 2.5 | 27.6 | ± | 0.9 | 50.4 | ± | 2.2 | 50.8 | ± | 10.5 | 101.2 | ± | 8.6 |
| Hyb 10 | 12.2 | ± | 0.2 | 6.3 | ± | 0.3 | 18.5 | ± | 0.4 | 30.8 | ± | 1.5 | 49.3 | ± | 1.9 | 92.2 | ± | 9.2 | 141.6 | ± | 9.5 |
| Hyb 11 | 18.1 | ± | 2.6 | 9.4 | ± | 1.2 | 27.5 | ± | 3.7 | 34.1 | ± | 1.7 | 61.7 | ± | 5.3 | 90.7 | ± | 11.7 | 152.3 | ± | 16.7 |
| Hyb 12 | 16.3 | ± | 1.4 | 6.7 | ± | 0.7 | 22.9 | ± | 2.0 | 34.0 | ± | 1.2 | 56.9 | ± | 1.3 | 96.2 | ± | 19.0 | 153.2 | ± | 17.7 |
| Hyb 13 | 9.0 | ± | 1.1 | 7.0 | ± | 0.7 | 16.0 | ± | 1.8 | 32.4 | ± | 1.5 | 48.4 | ± | 1.3 | 108.5 | ± | 7.1 | 156.9 | ± | 6.0 |
| Hyb 14 | 16.1 | ± | 1.9 | 7.0 | ± | 0.9 | 23.1 | ± | 2.8 | 36.6 | ± | 0.9 | 59.6 | ± | 3.6 | 130.1 | ± | 9.2 | 189.7 | ± | 5.6 |
| Hyb 15 | 14.8 | ± | 1.6 | 6.7 | ± | 0.9 | 21.4 | ± | 2.4 | 31.2 | ± | 0.1 | 52.6 | ± | 2.5 | 78.6 | ± | 0.2 | 131.3 | ± | 2.8 |
| Hyb 16 | 20.3 | ± | 3.1 | 5.7 | ± | 0.6 | 26.0 | ± | 3.6 | 29.9 | ± | 2.8 | 55.9 | ± | 0.8 | 86.1 | ± | 26.2 | 142.0 | ± | 25.3 |
| Hyb 17 | 15.7 | ± | 1.4 | 8.2 | ± | 0.8 | 23.9 | ± | 2.1 | 32.5 | ± | 1.6 | 56.4 | ± | 3.7 | 62.4 | ± | 7.6 | 118.8 | ± | 4.1 |
| Hyb 18 | 11.2 | ± | 1.4 | 5.2 | ± | 0.5 | 16.4 | ± | 1.9 | 29.4 | ± | 0.7 | 45.8 | ± | 1.4 | 99.3 | ± | 14.7 | 145.1 | ± | 13.4 |
| Hyb 19 | 13.5 | ± | 0.4 | 7.6 | ± | 0.2 | 21.1 | ± | 0.5 | 31.5 | ± | 1.0 | 52.6 | ± | 1.4 | 55.5 | ± | 5.7 | 108.2 | ± | 5.8 |
| Hyb 20 | 24.0 | ± | 2.4 | 9.9 | ± | 0.1 | 33.9 | ± | 2.5 | 34.2 | ± | 1.3 | 68.1 | ± | 1.2 | 46.2 | ± | 11.2 | 114.3 | ± | 10.2 |
| Hyb 21 | 7.7 | ± | 3.0 | 3.1 | ± | 0.4 | 10.9 | ± | 3.4 | 30.8 | ± | 1.1 | 41.7 | ± | 2.7 | 145.0 | ± | 18.4 | 186.7 | ± | 16.1 |
| Hyb 22 | 17.1 | ± | 1.7 | 7.7 | ± | 0.6 | 24.8 | ± | 2.1 | 40.3 | ± | 7.6 | 65.1 | ± | 9.3 | 133.6 | ± | 27.5 | 198.8 | ± | 35.8 |
| Hyb 23 | 14.0 | ± | 0.7 | 7.8 | ± | 0.7 | 21.8 | ± | 1.4 | 28.5 | ± | 1.7 | 50.3 | ± | 2.2 | 85.7 | ± | 8.3 | 136.0 | ± | 10.1 |
| Average: | 14.7 | ± | 1.0 | 6.9 | ± | 0.5 | 21.6 | ± | 1.4 | 32.0 | ± | 0.8 | 53.6 | ± | 1.8 | 87.8 | ± | 7.3 | 141.4 | ± | 7.0 |
| | | | | | | | | | | | | | | | | | | | | | |
| F Pr: | >.001 | | | >.001 | | | >.001 | | | 0.01 | | | >.001 | | | >.001 | | | >.001 | | |
b
