# Supplementary material for: Predicting future biomass yield in Miscanthus using the carbohydrate metabolic profile as a biomarker
Source: Glob Change Biol Bioenergy. 2017 Jan 21;9(7):1264–78. doi: 10.1111/gcbb.12418 (PMC5488626; doi:10.1111/gcbb.12418)
Supplement: Supplementary file 3 — Table S3. Cell wall composition in a mixed population (a) and mapping family (b). All carbohydrates are in mg g−1 DW. Statistics show differences between all genotypes from anova (P =≤ 0.05). N = 3, ±SE. [file GCBB-9-1264-s003.pptx]

## Slide 1
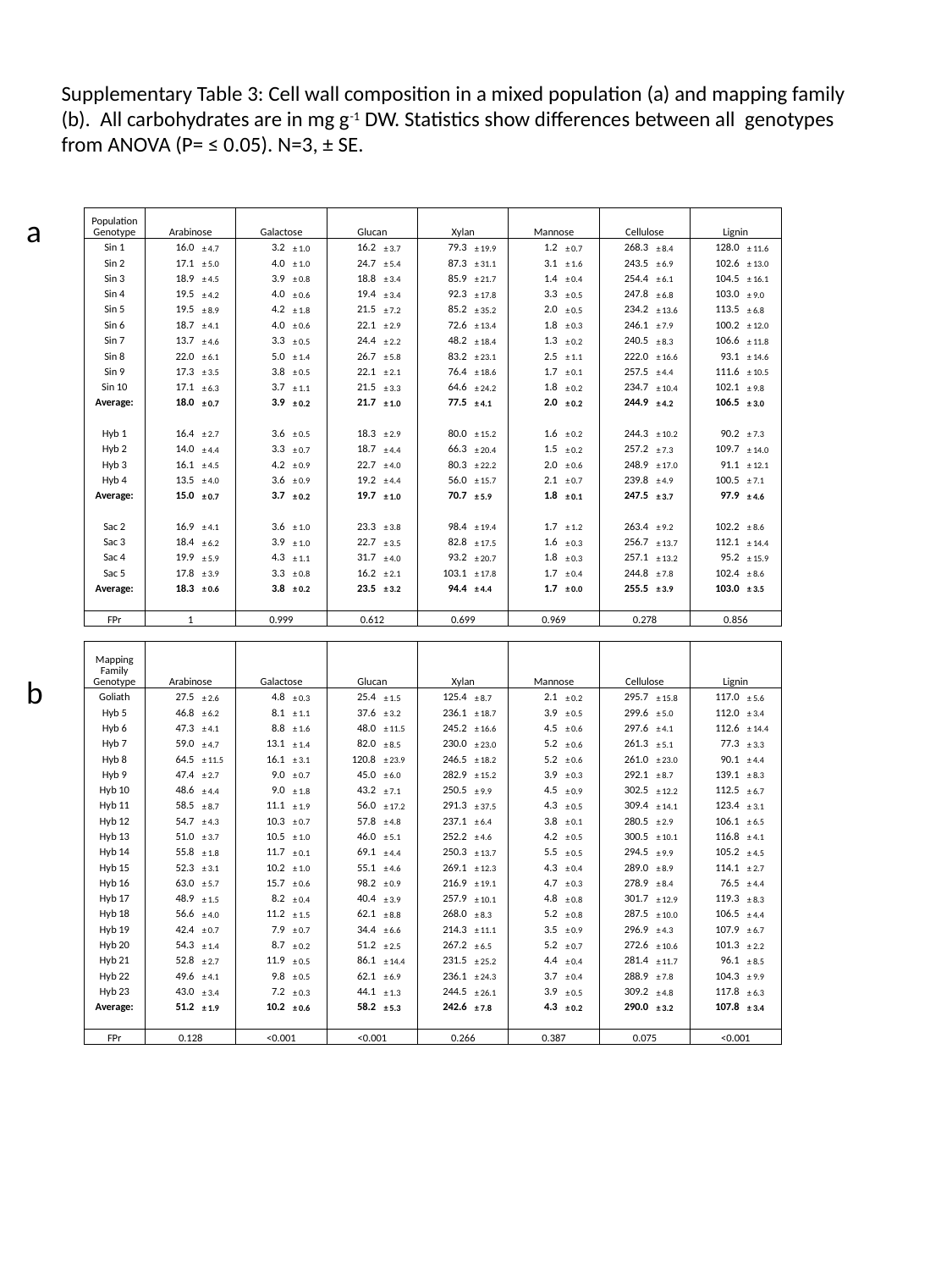

Supplementary Table 3: Cell wall composition in a mixed population (a) and mapping family (b). All carbohydrates are in mg g-1 DW. Statistics show differences between all genotypes from ANOVA (P= ≤ 0.05). N=3, ± SE.
a
| Population Genotype | Arabinose | | | Galactose | | | Glucan | | | Xylan | | | Mannose | | | Cellulose | | | Lignin | | |
| --- | --- | --- | --- | --- | --- | --- | --- | --- | --- | --- | --- | --- | --- | --- | --- | --- | --- | --- | --- | --- | --- |
| Sin 1 | 16.0 | ± | 4.7 | 3.2 | ± | 1.0 | 16.2 | ± | 3.7 | 79.3 | ± | 19.9 | 1.2 | ± | 0.7 | 268.3 | ± | 8.4 | 128.0 | ± | 11.6 |
| Sin 2 | 17.1 | ± | 5.0 | 4.0 | ± | 1.0 | 24.7 | ± | 5.4 | 87.3 | ± | 31.1 | 3.1 | ± | 1.6 | 243.5 | ± | 6.9 | 102.6 | ± | 13.0 |
| Sin 3 | 18.9 | ± | 4.5 | 3.9 | ± | 0.8 | 18.8 | ± | 3.4 | 85.9 | ± | 21.7 | 1.4 | ± | 0.4 | 254.4 | ± | 6.1 | 104.5 | ± | 16.1 |
| Sin 4 | 19.5 | ± | 4.2 | 4.0 | ± | 0.6 | 19.4 | ± | 3.4 | 92.3 | ± | 17.8 | 3.3 | ± | 0.5 | 247.8 | ± | 6.8 | 103.0 | ± | 9.0 |
| Sin 5 | 19.5 | ± | 8.9 | 4.2 | ± | 1.8 | 21.5 | ± | 7.2 | 85.2 | ± | 35.2 | 2.0 | ± | 0.5 | 234.2 | ± | 13.6 | 113.5 | ± | 6.8 |
| Sin 6 | 18.7 | ± | 4.1 | 4.0 | ± | 0.6 | 22.1 | ± | 2.9 | 72.6 | ± | 13.4 | 1.8 | ± | 0.3 | 246.1 | ± | 7.9 | 100.2 | ± | 12.0 |
| Sin 7 | 13.7 | ± | 4.6 | 3.3 | ± | 0.5 | 24.4 | ± | 2.2 | 48.2 | ± | 18.4 | 1.3 | ± | 0.2 | 240.5 | ± | 8.3 | 106.6 | ± | 11.8 |
| Sin 8 | 22.0 | ± | 6.1 | 5.0 | ± | 1.4 | 26.7 | ± | 5.8 | 83.2 | ± | 23.1 | 2.5 | ± | 1.1 | 222.0 | ± | 16.6 | 93.1 | ± | 14.6 |
| Sin 9 | 17.3 | ± | 3.5 | 3.8 | ± | 0.5 | 22.1 | ± | 2.1 | 76.4 | ± | 18.6 | 1.7 | ± | 0.1 | 257.5 | ± | 4.4 | 111.6 | ± | 10.5 |
| Sin 10 | 17.1 | ± | 6.3 | 3.7 | ± | 1.1 | 21.5 | ± | 3.3 | 64.6 | ± | 24.2 | 1.8 | ± | 0.2 | 234.7 | ± | 10.4 | 102.1 | ± | 9.8 |
| Average: | 18.0 | ± | 0.7 | 3.9 | ± | 0.2 | 21.7 | ± | 1.0 | 77.5 | ± | 4.1 | 2.0 | ± | 0.2 | 244.9 | ± | 4.2 | 106.5 | ± | 3.0 |
| | | | | | | | | | | | | | | | | | | | | | |
| Hyb 1 | 16.4 | ± | 2.7 | 3.6 | ± | 0.5 | 18.3 | ± | 2.9 | 80.0 | ± | 15.2 | 1.6 | ± | 0.2 | 244.3 | ± | 10.2 | 90.2 | ± | 7.3 |
| Hyb 2 | 14.0 | ± | 4.4 | 3.3 | ± | 0.7 | 18.7 | ± | 4.4 | 66.3 | ± | 20.4 | 1.5 | ± | 0.2 | 257.2 | ± | 7.3 | 109.7 | ± | 14.0 |
| Hyb 3 | 16.1 | ± | 4.5 | 4.2 | ± | 0.9 | 22.7 | ± | 4.0 | 80.3 | ± | 22.2 | 2.0 | ± | 0.6 | 248.9 | ± | 17.0 | 91.1 | ± | 12.1 |
| Hyb 4 | 13.5 | ± | 4.0 | 3.6 | ± | 0.9 | 19.2 | ± | 4.4 | 56.0 | ± | 15.7 | 2.1 | ± | 0.7 | 239.8 | ± | 4.9 | 100.5 | ± | 7.1 |
| Average: | 15.0 | ± | 0.7 | 3.7 | ± | 0.2 | 19.7 | ± | 1.0 | 70.7 | ± | 5.9 | 1.8 | ± | 0.1 | 247.5 | ± | 3.7 | 97.9 | ± | 4.6 |
| | | | | | | | | | | | | | | | | | | | | | |
| Sac 2 | 16.9 | ± | 4.1 | 3.6 | ± | 1.0 | 23.3 | ± | 3.8 | 98.4 | ± | 19.4 | 1.7 | ± | 1.2 | 263.4 | ± | 9.2 | 102.2 | ± | 8.6 |
| Sac 3 | 18.4 | ± | 6.2 | 3.9 | ± | 1.0 | 22.7 | ± | 3.5 | 82.8 | ± | 17.5 | 1.6 | ± | 0.3 | 256.7 | ± | 13.7 | 112.1 | ± | 14.4 |
| Sac 4 | 19.9 | ± | 5.9 | 4.3 | ± | 1.1 | 31.7 | ± | 4.0 | 93.2 | ± | 20.7 | 1.8 | ± | 0.3 | 257.1 | ± | 13.2 | 95.2 | ± | 15.9 |
| Sac 5 | 17.8 | ± | 3.9 | 3.3 | ± | 0.8 | 16.2 | ± | 2.1 | 103.1 | ± | 17.8 | 1.7 | ± | 0.4 | 244.8 | ± | 7.8 | 102.4 | ± | 8.6 |
| Average: | 18.3 | ± | 0.6 | 3.8 | ± | 0.2 | 23.5 | ± | 3.2 | 94.4 | ± | 4.4 | 1.7 | ± | 0.0 | 255.5 | ± | 3.9 | 103.0 | ± | 3.5 |
| | | | | | | | | | | | | | | | | | | | | | |
| FPr | 1 | | | 0.999 | | | 0.612 | | | 0.699 | | | 0.969 | | | 0.278 | | | 0.856 | | |
| | | | | | | | | | | | | | | | | | | | | | |
| Mapping Family Genotype | Arabinose | | | Galactose | | | Glucan | | | Xylan | | | Mannose | | | Cellulose | | | Lignin | | |
| Goliath | 27.5 | ± | 2.6 | 4.8 | ± | 0.3 | 25.4 | ± | 1.5 | 125.4 | ± | 8.7 | 2.1 | ± | 0.2 | 295.7 | ± | 15.8 | 117.0 | ± | 5.6 |
| Hyb 5 | 46.8 | ± | 6.2 | 8.1 | ± | 1.1 | 37.6 | ± | 3.2 | 236.1 | ± | 18.7 | 3.9 | ± | 0.5 | 299.6 | ± | 5.0 | 112.0 | ± | 3.4 |
| Hyb 6 | 47.3 | ± | 4.1 | 8.8 | ± | 1.6 | 48.0 | ± | 11.5 | 245.2 | ± | 16.6 | 4.5 | ± | 0.6 | 297.6 | ± | 4.1 | 112.6 | ± | 14.4 |
| Hyb 7 | 59.0 | ± | 4.7 | 13.1 | ± | 1.4 | 82.0 | ± | 8.5 | 230.0 | ± | 23.0 | 5.2 | ± | 0.6 | 261.3 | ± | 5.1 | 77.3 | ± | 3.3 |
| Hyb 8 | 64.5 | ± | 11.5 | 16.1 | ± | 3.1 | 120.8 | ± | 23.9 | 246.5 | ± | 18.2 | 5.2 | ± | 0.6 | 261.0 | ± | 23.0 | 90.1 | ± | 4.4 |
| Hyb 9 | 47.4 | ± | 2.7 | 9.0 | ± | 0.7 | 45.0 | ± | 6.0 | 282.9 | ± | 15.2 | 3.9 | ± | 0.3 | 292.1 | ± | 8.7 | 139.1 | ± | 8.3 |
| Hyb 10 | 48.6 | ± | 4.4 | 9.0 | ± | 1.8 | 43.2 | ± | 7.1 | 250.5 | ± | 9.9 | 4.5 | ± | 0.9 | 302.5 | ± | 12.2 | 112.5 | ± | 6.7 |
| Hyb 11 | 58.5 | ± | 8.7 | 11.1 | ± | 1.9 | 56.0 | ± | 17.2 | 291.3 | ± | 37.5 | 4.3 | ± | 0.5 | 309.4 | ± | 14.1 | 123.4 | ± | 3.1 |
| Hyb 12 | 54.7 | ± | 4.3 | 10.3 | ± | 0.7 | 57.8 | ± | 4.8 | 237.1 | ± | 6.4 | 3.8 | ± | 0.1 | 280.5 | ± | 2.9 | 106.1 | ± | 6.5 |
| Hyb 13 | 51.0 | ± | 3.7 | 10.5 | ± | 1.0 | 46.0 | ± | 5.1 | 252.2 | ± | 4.6 | 4.2 | ± | 0.5 | 300.5 | ± | 10.1 | 116.8 | ± | 4.1 |
| Hyb 14 | 55.8 | ± | 1.8 | 11.7 | ± | 0.1 | 69.1 | ± | 4.4 | 250.3 | ± | 13.7 | 5.5 | ± | 0.5 | 294.5 | ± | 9.9 | 105.2 | ± | 4.5 |
| Hyb 15 | 52.3 | ± | 3.1 | 10.2 | ± | 1.0 | 55.1 | ± | 4.6 | 269.1 | ± | 12.3 | 4.3 | ± | 0.4 | 289.0 | ± | 8.9 | 114.1 | ± | 2.7 |
| Hyb 16 | 63.0 | ± | 5.7 | 15.7 | ± | 0.6 | 98.2 | ± | 0.9 | 216.9 | ± | 19.1 | 4.7 | ± | 0.3 | 278.9 | ± | 8.4 | 76.5 | ± | 4.4 |
| Hyb 17 | 48.9 | ± | 1.5 | 8.2 | ± | 0.4 | 40.4 | ± | 3.9 | 257.9 | ± | 10.1 | 4.8 | ± | 0.8 | 301.7 | ± | 12.9 | 119.3 | ± | 8.3 |
| Hyb 18 | 56.6 | ± | 4.0 | 11.2 | ± | 1.5 | 62.1 | ± | 8.8 | 268.0 | ± | 8.3 | 5.2 | ± | 0.8 | 287.5 | ± | 10.0 | 106.5 | ± | 4.4 |
| Hyb 19 | 42.4 | ± | 0.7 | 7.9 | ± | 0.7 | 34.4 | ± | 6.6 | 214.3 | ± | 11.1 | 3.5 | ± | 0.9 | 296.9 | ± | 4.3 | 107.9 | ± | 6.7 |
| Hyb 20 | 54.3 | ± | 1.4 | 8.7 | ± | 0.2 | 51.2 | ± | 2.5 | 267.2 | ± | 6.5 | 5.2 | ± | 0.7 | 272.6 | ± | 10.6 | 101.3 | ± | 2.2 |
| Hyb 21 | 52.8 | ± | 2.7 | 11.9 | ± | 0.5 | 86.1 | ± | 14.4 | 231.5 | ± | 25.2 | 4.4 | ± | 0.4 | 281.4 | ± | 11.7 | 96.1 | ± | 8.5 |
| Hyb 22 | 49.6 | ± | 4.1 | 9.8 | ± | 0.5 | 62.1 | ± | 6.9 | 236.1 | ± | 24.3 | 3.7 | ± | 0.4 | 288.9 | ± | 7.8 | 104.3 | ± | 9.9 |
| Hyb 23 | 43.0 | ± | 3.4 | 7.2 | ± | 0.3 | 44.1 | ± | 1.3 | 244.5 | ± | 26.1 | 3.9 | ± | 0.5 | 309.2 | ± | 4.8 | 117.8 | ± | 6.3 |
| Average: | 51.2 | ± | 1.9 | 10.2 | ± | 0.6 | 58.2 | ± | 5.3 | 242.6 | ± | 7.8 | 4.3 | ± | 0.2 | 290.0 | ± | 3.2 | 107.8 | ± | 3.4 |
| | | | | | | | | | | | | | | | | | | | | | |
| FPr | 0.128 | | | <0.001 | | | <0.001 | | | 0.266 | | | 0.387 | | | 0.075 | | | <0.001 | | |
b
