# Supplementary material for: Predicting future biomass yield in Miscanthus using the carbohydrate metabolic profile as a biomarker
Source: Glob Change Biol Bioenergy. 2017 Jan 21;9(7):1264–78. doi: 10.1111/gcbb.12418 (PMC5488626; doi:10.1111/gcbb.12418)
Supplement: Supplementary file 4 — Table S4. Block effects across the two trials. For the mixed population N = 18 and for the mapping family N = 19. Statistics (F Pr) show the results of a one‐way anova with block as a treatment factor (Significant differences = ≤ 0.05). [file GCBB-9-1264-s004.pptx]

## Slide 1
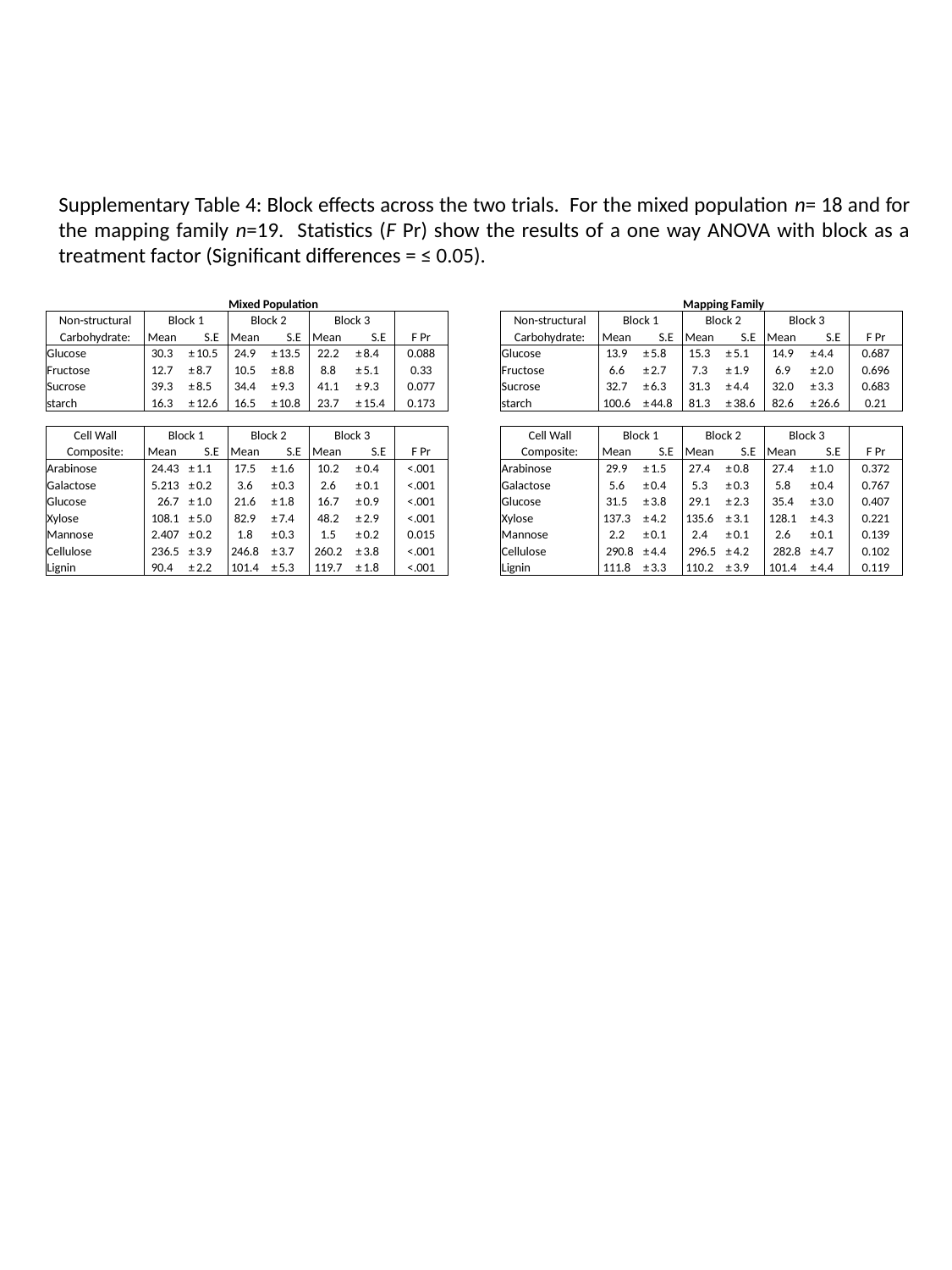

Supplementary Table 4: Block effects across the two trials. For the mixed population n= 18 and for the mapping family n=19. Statistics (F Pr) show the results of a one way ANOVA with block as a treatment factor (Significant differences = ≤ 0.05).
| | | | | Mixed Population | | | | | | | | | | | | Mapping Family | | | | | | |
| --- | --- | --- | --- | --- | --- | --- | --- | --- | --- | --- | --- | --- | --- | --- | --- | --- | --- | --- | --- | --- | --- | --- |
| Non-structural | Block 1 | | | Block 2 | | | Block 3 | | | | | Non-structural | Block 1 | | | Block 2 | | | Block 3 | | | |
| Carbohydrate: | Mean | | S.E | Mean | | S.E | Mean | | S.E | F Pr | | Carbohydrate: | Mean | | S.E | Mean | | S.E | Mean | | S.E | F Pr |
| Glucose | 30.3 | ± | 10.5 | 24.9 | ± | 13.5 | 22.2 | ± | 8.4 | 0.088 | | Glucose | 13.9 | ± | 5.8 | 15.3 | ± | 5.1 | 14.9 | ± | 4.4 | 0.687 |
| Fructose | 12.7 | ± | 8.7 | 10.5 | ± | 8.8 | 8.8 | ± | 5.1 | 0.33 | | Fructose | 6.6 | ± | 2.7 | 7.3 | ± | 1.9 | 6.9 | ± | 2.0 | 0.696 |
| Sucrose | 39.3 | ± | 8.5 | 34.4 | ± | 9.3 | 41.1 | ± | 9.3 | 0.077 | | Sucrose | 32.7 | ± | 6.3 | 31.3 | ± | 4.4 | 32.0 | ± | 3.3 | 0.683 |
| starch | 16.3 | ± | 12.6 | 16.5 | ± | 10.8 | 23.7 | ± | 15.4 | 0.173 | | starch | 100.6 | ± | 44.8 | 81.3 | ± | 38.6 | 82.6 | ± | 26.6 | 0.21 |
| | | | | | | | | | | | | | | | | | | | | | | |
| Cell Wall | Block 1 | | | Block 2 | | | Block 3 | | | | | Cell Wall | Block 1 | | | Block 2 | | | Block 3 | | | |
| Composite: | Mean | | S.E | Mean | | S.E | Mean | | S.E | F Pr | | Composite: | Mean | | S.E | Mean | | S.E | Mean | | S.E | F Pr |
| Arabinose | 24.43 | ± | 1.1 | 17.5 | ± | 1.6 | 10.2 | ± | 0.4 | <.001 | | Arabinose | 29.9 | ± | 1.5 | 27.4 | ± | 0.8 | 27.4 | ± | 1.0 | 0.372 |
| Galactose | 5.213 | ± | 0.2 | 3.6 | ± | 0.3 | 2.6 | ± | 0.1 | <.001 | | Galactose | 5.6 | ± | 0.4 | 5.3 | ± | 0.3 | 5.8 | ± | 0.4 | 0.767 |
| Glucose | 26.7 | ± | 1.0 | 21.6 | ± | 1.8 | 16.7 | ± | 0.9 | <.001 | | Glucose | 31.5 | ± | 3.8 | 29.1 | ± | 2.3 | 35.4 | ± | 3.0 | 0.407 |
| Xylose | 108.1 | ± | 5.0 | 82.9 | ± | 7.4 | 48.2 | ± | 2.9 | <.001 | | Xylose | 137.3 | ± | 4.2 | 135.6 | ± | 3.1 | 128.1 | ± | 4.3 | 0.221 |
| Mannose | 2.407 | ± | 0.2 | 1.8 | ± | 0.3 | 1.5 | ± | 0.2 | 0.015 | | Mannose | 2.2 | ± | 0.1 | 2.4 | ± | 0.1 | 2.6 | ± | 0.1 | 0.139 |
| Cellulose | 236.5 | ± | 3.9 | 246.8 | ± | 3.7 | 260.2 | ± | 3.8 | <.001 | | Cellulose | 290.8 | ± | 4.4 | 296.5 | ± | 4.2 | 282.8 | ± | 4.7 | 0.102 |
| Lignin | 90.4 | ± | 2.2 | 101.4 | ± | 5.3 | 119.7 | ± | 1.8 | <.001 | | Lignin | 111.8 | ± | 3.3 | 110.2 | ± | 3.9 | 101.4 | ± | 4.4 | 0.119 |
