# Supplementary material for: Predicting future biomass yield in Miscanthus using the carbohydrate metabolic profile as a biomarker
Source: Glob Change Biol Bioenergy. 2017 Jan 21;9(7):1264–78. doi: 10.1111/gcbb.12418 (PMC5488626; doi:10.1111/gcbb.12418)
Supplement: Supplementary file 5 — Table S5. Carbohydrate predictors of biomass traits selected by CfsSubsetEval (in the Weka software) for each of the machine learning models in Table 2. [file GCBB-9-1264-s005.docx]

Supplementary Table 5: Carbohydrate predictors of biomass traits selected by *CfsSubsetEval* (in the Weka software) for each of the machine learning models in Table 2.

**Height**

|  | | Information included in models | | |
| --- | --- | --- | --- | --- |
|  |  | Genotype, carbohydrates | Carbohydrates only | |
|  |  | All genotype replicates | All genotype replicates | Averaged by genotype |
| Plants | Carbohydrate fractions | Predictors selected by *CfsSubsetEval* | | |
| Mixed population | All | Fru, Glucan, Hex, Sta | Fru, Glucan, Hex, Sta | Ara, Fru, Gal, Glucan, Sta/Glc |
|  | NSC | Fru, Hex, Sta, Sta/Glc | Fru, Hex, Sta, Suc/Glc | Fru, Hex, Sta |
|  | Soluble | Fru, Suc/Glc | Fru, Suc/Glc | Fru, Hex, Suc/Glc |
| Mapping family 2013 | All | Ara, Fru, Glc/Fru, Glucan, Lig, Suc, Suc/Fru, Suc/Sta | Ara, Fru, Glc/Fru, Glucan, Lig, Suc, Suc/Fru, Suc/Sta | Ara, Cel, Glc/Fru, Glucan, Lig, Suc, Suc/Sta |
|  | NSC | Glc/Fru, NSC, Suc/Fru | Glc/Fru, NSC, Suc/Fru | Fru, Glc/Fru, Suc, Suc/Fru, Suc/Sta |
|  | Soluble | Glc/Fru, Suc, Suc/Fru | Glc/Fru, Suc, Suc/Fru | Fru, Glc/Fru, Suc, Suc/Fru |
| Mapping family 2014 | NCS | Glc/Fru, Suc/Fru, Suc/Sta | Glc/Fru, Suc/Fru, Suc/Sta | Fru, Glc/Fru, Suc, Suc/Sta |
|  | Soluble | Fru, Glc/Fru | Fru, Glc/Fru | Fru, Glc/Fru, Suc |

**Harvest yield**

aaa

|  | | Information included in models | | |
| --- | --- | --- | --- | --- |
|  |  | Genotype, carbohydrates | Carbohydrates only | |
|  |  | All genotype replicates | All genotype replicates | Averaged by genotype |
| Plants | Carbohydrate fractions | Predictors selected by *CfsSubsetEval* | | |
| Mixed population | All | Fru, Gal, Suc/Glc | Fru, Gal, Suc/Glc | Ara, Fru |
|  | NSC | Fru, Suc/Glc | Fru, Suc/Glc | Fru, Suc |
|  | Soluble | Fru, Suc/Glc | Fru, Suc/Glc | Fru, Suc |
| Mapping family 2013 | All | Ara, Cel, Fru, Glc/Fru, Glucan, Lig, NSC | Ara, Cel, Fru, Glc/Fru, Glucan, Lig, NSC | Ara, Cel, Glc/Fru, Glucan, Lig, Man, NSC |
|  | NSC | Glc/Fru, NSC, Suc/Fru | Glc/Fru, NSC, Suc/Fru | Glc/Fru, NSC, Suc/Fru |
|  | Soluble | Glc/Fru, Suc, Suc/Fru | Glc/Fru, Suc, Suc/Fru | Glc/Fru, Suc, Suc/Fru |
| Mapping family 2014 | NSC | Glc/Fru, Suc/Fru, Suc/Sta | Glc/Fru, Suc/Fru, Suc/Sta | Glc/Fru, Suc/Fru, Suc/Sta |
|  | Soluble | Glc/Fru, Suc/Fru | Glc/Fru, Suc/Fru | Fru, Glc/Fru |
